# Contribution of Respiratory Syncytial Virus to Burden of Lower Respiratory Tract Infections: A Global Analysis of 204 Countries and Territories, 1990–2021

**DOI:** 10.3390/tropicalmed10080223

**Published:** 2025-08-11

**Authors:** Zhiwei Chen, Qiu Zhang, Junrong Li, Naihong Xie, Qingmei Zheng, Youzhen Lai, Xiaoyang Zhang

**Affiliations:** 1 The Affiliated Fuzhou Center for Disease Control and Prevention of Fujian Medical University, Fuzhou 350209, China; zerochen98@hotmail.com (Z.C.); zhqiu811021@outlook.com (Q.Z.); icedblue718@hotmail.com (N.X.); zhengqingmei93@hotmail.com (Q.Z.); laiyouzhen@hotmail.com (Y.L.); 2 The School of Public Health, Fujian Medical University, Fuzhou 350012, China; 3 Fujian Provincial Center for Disease Control and Prevention, Fuzhou 350012, China; junrong_li@hotmail.com

**Keywords:** global burden of disease, respiratory syncytial virus, lower respiratory tract infections, socio-demographic index, disability-adjusted life years

## Abstract

Respiratory syncytial virus (RSV) is a major cause of morbidity and mortality from lower respiratory infections (LRIs) worldwide. This study analyzes trends in age-standardized death rates (ASDRs) and disability-adjusted life years (DALYs) due to RSV-induced LRIs from 1990 to 2019, using data from the Global Burden of Disease Study 2021 (GBD 2021). The findings show a gradual decline in deaths, ASDR, and DALYs throughout this period. However, these indicators were higher in men than in women, as well as more pronounced in sub-Saharan West Africa. Developed countries exhibited higher ASDR and DALY values than developing countries, with the highest burden observed among children and the elderly in low Socio-demographic Index (SDI) regions. Globally, RSV-induced LRIs have shown a significant reduction in burden, but interventions are still urgently needed—particularly in low SDI areas—to reduce the impact on vulnerable populations. Public health policies focusing on these high-risk groups are essential for addressing the remaining disparities in RSV-related morbidity and mortality.

## 1. Introduction

Respiratory syncytial virus (RSV) is one of the most common pathogens responsible for lower respiratory infections worldwide. RSV infections are a leading cause of acute bronchitis, pneumonia, and respiratory failure, particularly in infants, young children, the elderly, and immunocompromised individuals [1,2]. RSV infections typically exhibit high prevalence during the fall and winter seasons. The primary modes of transmission are droplet transmission and direct contact, and the infection can quickly spread to the lower respiratory tract, resulting in airway inflammation and respiratory obstruction, which pose significant health risks [3]. In infants and young children, RSV is a major cause of hospitalization and mortality [4]. In the elderly, RSV infections often lead to more severe clinical outcomes due to the weakening of the immune system [5]. Therefore, RSV infections represent a significant public health concern.

RSV infections not only pose a threat to individual health but also represent a significant social and medical burden. Each year, RSV leads to the hospitalization of millions of infants, children, and elderly individuals worldwide, placing a substantial strain on healthcare resources. In low-income countries and regions, RSV infections are associated with higher mortality and hospitalization rates, which are closely linked to the scarcity of healthcare resources, low vaccination coverage, and insufficient public health infrastructure [6,7]. Moreover, RSV-related long-term health complications threaten patients’ quality of life, particularly in children who may develop persistent respiratory conditions such as asthma or recurrent pneumonia following infection [8]. These long-term effects not only increase healthcare costs but also exacerbate the strain on social security systems, especially in economically disadvantaged regions where the burden of RSV is particularly high [9]. Globally, seasonal RSV epidemics pose a significant challenge to public health systems. During peak winter months, hospitals and emergency departments often face overwhelming pressure, leading to shortages of healthcare resources. While high-income countries have achieved some success through vaccination and improved medical interventions, low-income countries continue to face more severe public health challenges [10,11,12]. Therefore, it is crucial to examine the latest epidemiological trends of RSV infections and to optimize prevention and control strategies to address the substantial burden on public health.

Although several studies have assessed the disease burden of RSV, comprehensive analyses of its global epidemiological trends, regional variations, and impacts on different populations require timely and systematic updates. Using data from the Global Burden of Disease (GBD) 2021 study, this study aims to thoroughly evaluate the burden of lower respiratory tract infections caused by RSV between 1990 and 2021 and to identify trends in the disease burden over this period. This study utilized ASDRs and DALYs to measure the impact of RSV cases. ASDRs were chosen for their ability to standardize death rates across different age structures, providing a clear picture of mortality impact. DALYs were selected as they combine years of life lost due to premature mortality (YLLs) and years lived with disability (YLDs), offering a comprehensive assessment of both mortality and morbidity burdens. Together, these metrics enable a more complete understanding of the disease’s impact and facilitate comparisons across different populations and regions. Additionally, the differences in burden across regions, genders, and age groups were analyzed, and future projections were made. By analyzing the global burden of RSV, we seek to provide more accurate insights to support global public health decision-making, particularly in regions with low SDI and among key populations, while also offering a scientific foundation for the development of future vaccination and intervention strategies.

## 2. Materials and Methods

### 2.1. Data Source

The GBD 2021 study represents a comprehensive compilation of epidemiological data from 204 countries and territories worldwide. It covers morbidity and mortality for 288 causes of death, as well as the analysis of health losses associated with 369 diseases and 88 risk factors. The primary data sources include population-based registries and cause-of-death inference systems, providing a thorough overview of all 288 causes of death. Additionally, the study incorporates data from surveys, censuses, surveillance systems, and cancer registries to facilitate more detailed analyses. Police records, open data sources, and minimally invasive tissue sampling were also employed to gather disease- and injury-specific data. During data processing and estimation, the GBD 2021 team utilized advanced technological tools, such as the Cause of Death Integrated Model (CODEm), to ensure the accuracy and reliability of the results. In instances where data were insufficient or exhibited unusual epidemiological patterns, alternative modeling and estimation methods were applied. For example, when cancer registries had limited coverage or lacked reliable mortality data, GBD 2021 relied on predictive modeling, data sharing, and expert consultation to estimate missing data.

This study utilized data on lower respiratory infections caused by respiratory syncytial virus (RSV), sourced from the Global Health Data Exchange (GHDx) website (https://vizhub.healthdata.org/gbd-results/, accessed on 20 December 2024). We extracted annual statistics on morbidity, mortality, DALYs, and their corresponding age-standardized rates (ASRs) for LRIs attributable to RSV exposure between 1990 and 2019.

Morbidity and mortality from lower respiratory infections caused by RSV were identified using the International Classification of Diseases, Ninth (ICD-9) and Tenth (ICD-10) editions. Specifically, RSV-associated LRI cases are defined through ICD-10 codes, including related codes such as J20-J22 and J40-J47. In the case of ICD-9, the corresponding codes include categories such as 460–466 and 480–487. We extracted estimates for prevalence, incidence, and disability-adjusted life years (DALYs) related to RSV-induced LRIs from the GBD 2021 database and obtained the corresponding 95% uncertainty intervals (UIs). The calculation of DALYs involves summing the years lived with the disease and the years of life lost to assess the burden of RSV-induced LRIs. The Socio-demographic Index is a measure of socio-economic development in a country or region, based on indicators such as fertility rates, educational attainment, and per capita income. The SDI value ranges from 0 to 1, with higher values indicating greater socio-economic development. The GBD 2021 study classified countries and the 21 geographic regions into the following five tiers based on the SDI: High, Medium-High, Medium, Medium-Low, and Low.

### 2.2. Construction of the ARIMA Model

To project future trends in RSV-related LRI burden, we employed seasonal ARIMA (AutoRegressive Integrated Moving Average) models. The ARIMA model is widely used in time series prediction, and it has the following three key components: Autoregression (AR), which captures the relationship between an observation and its prior values (lags); Differencing (I), which stabilizes non-stationary data by removing trends (e.g., via first- or second-order differencing); and Moving Average (MA), which models errors as a linear combination of past error terms.

The general form of the model is ARIMA(p, d, q), where p represents the order of the autoregressive terms (selected via partial autocorrelation plots); d represents the degree of differencing (tested using Augmented Dickey–Fuller tests for stationarity); and q represents the order of the moving average terms (identified via autocorrelation plots).

For seasonal trends (e.g., annual fluctuations in RSV incidence), we extended the model to SARIMA(p, d, q)(P, D, Q)[s], where s represents the seasonal period. Model parameters were optimized using the Akaike Information Criterion (AIC), and residuals were checked for white noise (randomness) via Ljung–Box tests. The seasonal model can be mathematically represented as follows:

$$\phi_{p}(B)\tilde{\phi_{p}}{(B}^{s})y_{t}^{*}=\theta_{p}(B)\tilde{\theta_{p}}{(B}^{s})\varepsilon_{t}$$

where $\phi_{p}(B)$ represents a non-seasonal autoregressive lag polynomial; $\tilde{\phi_{p}}{(B}^{s})$ represents seasonal moving average lag polynomial; and $\theta_{p}(B)$ represents seasonal moving average lag polynomial. To ensure the stability of our time series, we initially applied differencing, a crucial step in the analysis. We then conducted an augmented Dickey–Fuller (ADF) test to verify the temporal stability of the series. Subsequently, we employed the corrected Akaike’s information criterion (AICc) to assess the goodness of fit of the SARIMA model, with the model associated with the lowest AICc value considered the optimal choice. Finally, we conducted the Ljung–Box test to ascertain whether the residual sequence of the model exhibited characteristics of white noise. If the *p*-value is greater than 0.05, the model satisfies the test’s criteria and can be employed for predictive analysis.

### 2.3. Statistical Analysis

The methodology and protocols of the Global Burden of Disease (GBD) study have been described in detail in the previous literature [13]. In this study, we utilized annual incidence rates, mortality rates, DALYs, and their corresponding ASRs per 100,000 population to analyze the distribution of the burden of lower respiratory infections due to RSV exposure across different age and sex groups. Temporal trends in RSV-induced LRI were quantified using the ASR, DALY, and the estimated annual percentage change (EAPC). The ASR per 100,000 population was calculated as follows:

$$\mathrm{ASR}=\frac{\sum_{i=1}^{A}\alpha_{i}w_{i}}{\sum_{i=1}^{A}w_{i}}\;\times \;10000$$

where $\alpha_{i}$ represents the age-specific rate in the ith age group; w represents the number of people in the corresponding ith age group among the standard population; and A represents the number of age groups.

The Estimated Annual Percentage Change is commonly used in epidemiological studies to assess the time trend of disease ASRs. The EAPC coefficient, denoted as β, is derived from the natural logarithm of the ASR. In this study, β represents ln(ASR), while t denotes the calendar year. EAPC and its 95% confidence interval (CI) were calculated using the following linear regression model:

γ=α+βx+ε

EAPC=100 × (exp(β) − 1)

where

γ: ln(ASR) (the dependent variable, representing the natural log-transformed rate);

x: calendar year (the independent variable, representing time);

α: intercept of the regression line;

β: slope coefficient (annual rate of change in ln(ASR));

ε: random error term (residuals).

Subsequently, the EAPC was calculated as 100 × (exp(β) − 1). Positive values of the EAPC and its 95% CI indicate an increasing trend in ASRs, while negative values and their corresponding confidence intervals suggest a decreasing trend. Additionally, we used Pearson’s correlation coefficient (r) to examine the correlation between the age-standardized incidence rate (ASIR), the ASDR, and the Socio-demographic Index (SDI) to analyze the impact of socioeconomic factors on the burden of lower respiratory tract infections caused by RSV. The calculation of Pearson’s correlation coefficient accounted for significant differences in population size and case frequency across countries and regions, as detailed in the comprehensive analysis of the GBD 2021 study. For this calculation, age-standardized methods were applied, and raw data were converted to ASRs through weighted averaging to ensure equitable representation of each group. Pearson’s correlation coefficients were computed using the product–moment correlation formula, a method that appropriately considers statistical efficacy and precision while avoiding erroneous causal inferences. Furthermore, the study determined the confidence intervals for these correlation coefficients and performed sensitivity analyses to assess the stability of the results and to evaluate the potential effects of data bias and quality inconsistencies, thereby ensuring the robustness and reliability of the global health data analysis.

## 3. Results

### 3.1. Death and DALY Burden of Respiratory Syncytial Virus

Globally, the number of deaths from lower respiratory infections due to respiratory syncytial virus (RSV) exposure declined from 139,762 (95%UI: 123,666 to 158,110) in 1990 to 31,525 (95%UI: 23,348 to 41,871) in 2021. The ASDR also decreased from an estimated 2.31 (95%UI: 2.05 to 2.60) in 1990 to 0.49 (95%UI: 0.36 to 0.65) in 2021, with an EAPC of −2.62 (95% CI: −3.33 to −1.91) (Table 1). Meanwhile, DALYs demonstrated a clear downward trend, decreasing from 12,105,847 (95% CI: 10,665,949 to 13,740,784) in 1990 to 2,591,507 (95% CI: 1,902,003 to 3,468,792) in 2021. The age-standardized DALYs also decreased from 193.49 (95% CI: 170.61 to 219.46) to 40.82 (95% CI: 29.91 to 54.59), with an EAPC of −2.77 (95% CI: −3.44 to −2.09) (Table 2).

The number of deaths decreased in all SDI regions, with the greatest decreases in the high-medium SDI regions, with EAPCs of −5.24 (95% CI: −6.30 to −4.16) and −6.34 (95% CI: −7.34 to −5.33) for the ASDR and the age-standardized DALY rate, respectively (Table 1). Both the ASDR and the age-standardized DALY rate were significantly lower in high SDI areas, with EAPCs of −3.19 (95% CI: −4.84 to −1.51) and −3.38 (95% CI: −4.85 to −1.88), respectively, showing a strong downward trend. However, the rate of decline was relatively slower in the middle SDI, low-middle SDI, and low SDI regions, with relatively high EAPC values, although the ASDR and the age-standardized DALY rate also decreased (Table 2).

In terms of regional observations, Sub-Saharan West Africa recorded the highest number of deaths in 2021, totaling 41,103 (95% CI: 34,240–48,132). South Asia reported the highest number of DALYs at 959,423 (95% CI: 484,294–1,553,206). High-income Asia-Pacific had the lowest number of deaths and DALYs, with 2 (95% CI: 0–12) and 32 (95% CI: 3–167), respectively. Sub-Saharan West Africa also had the highest ASDR of 1.34 (95% CI: 0.80–2.00) and age-standardized DALY rate of 108.55 (95% CI: 63.85–162.60). In contrast, high-income Asia-Pacific recorded the lowest ASDR of 0.00 (95% CI: 0.00–0.00) and age-standardized DALY rate of 0.01 (95% CI: 0.00–0.06). From 1990 to 2021, East Asia exhibited the largest decline in ASDR, with an EAPC of −7.31 (95% CI: −8.16 to −6.45), while Sub-Saharan West Africa showed the smallest decline, with an EAPC of −1.50 (95% CI: −3.34 to 0.37). Similarly, East Asia experienced the greatest reduction in age-standardized DALY rates, with an EAPC of −8.14 (95% CI: −8.98 to −7.30), whereas Sub-Saharan West Africa had the least decline, with an EAPC of −1.68 (95% CI: −3.51 to 0.18) (Table 1 and Table 2).

**Figure 1:**
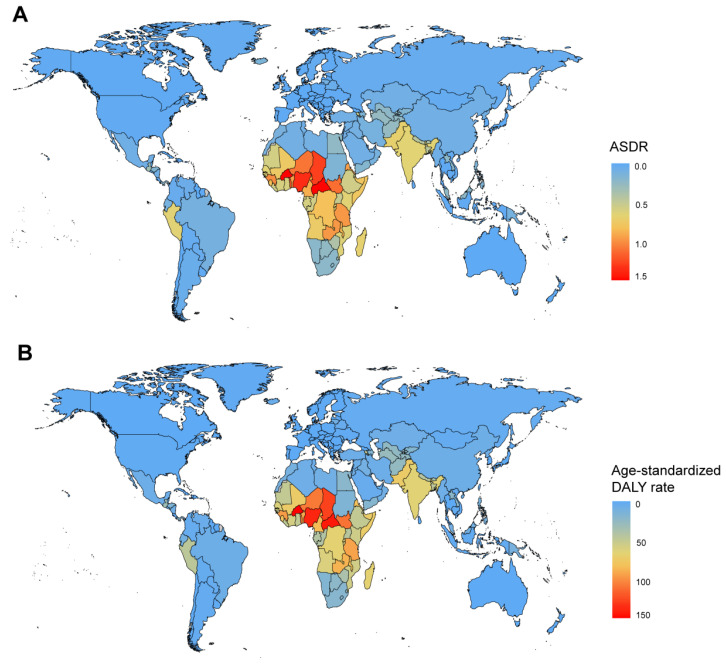
Age-standardized rates of RSV-related LRI in 204 countries or territories in 2021: (**A**) ASDR of 204 countries or territories in 2021; (**B**) age-standardized DALY rate of 204 countries or territories in 2021. RSV: respiratory syncytial virus; LRI: lower respiratory infections; ASDR: age-standardized death rate; DALYs: disability-adjusted life years.

**Figure 2:**
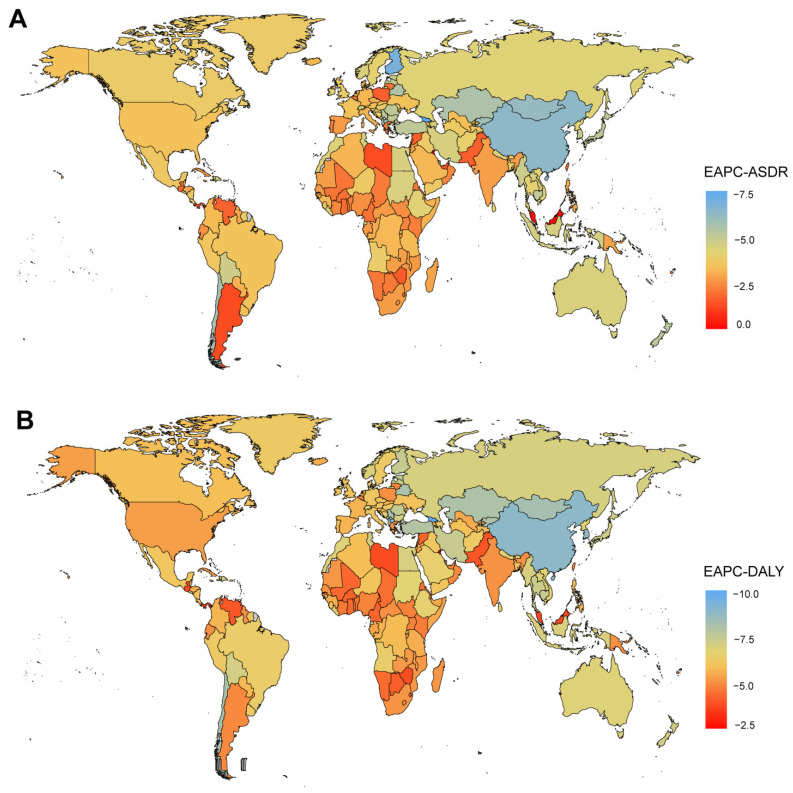
Global EAPCs of RSV-related LRI in 204 countries or territories in 2021: (**A**) EAPCs of deaths in 2021; (**B**) EAPCs of DALYs in 2021. RSV: respiratory syncytial virus; LRI: lower respiratory infections; DALYs: disability-adjusted life years; EAPCs: estimated annual percentage changes.

**Figure 3:**
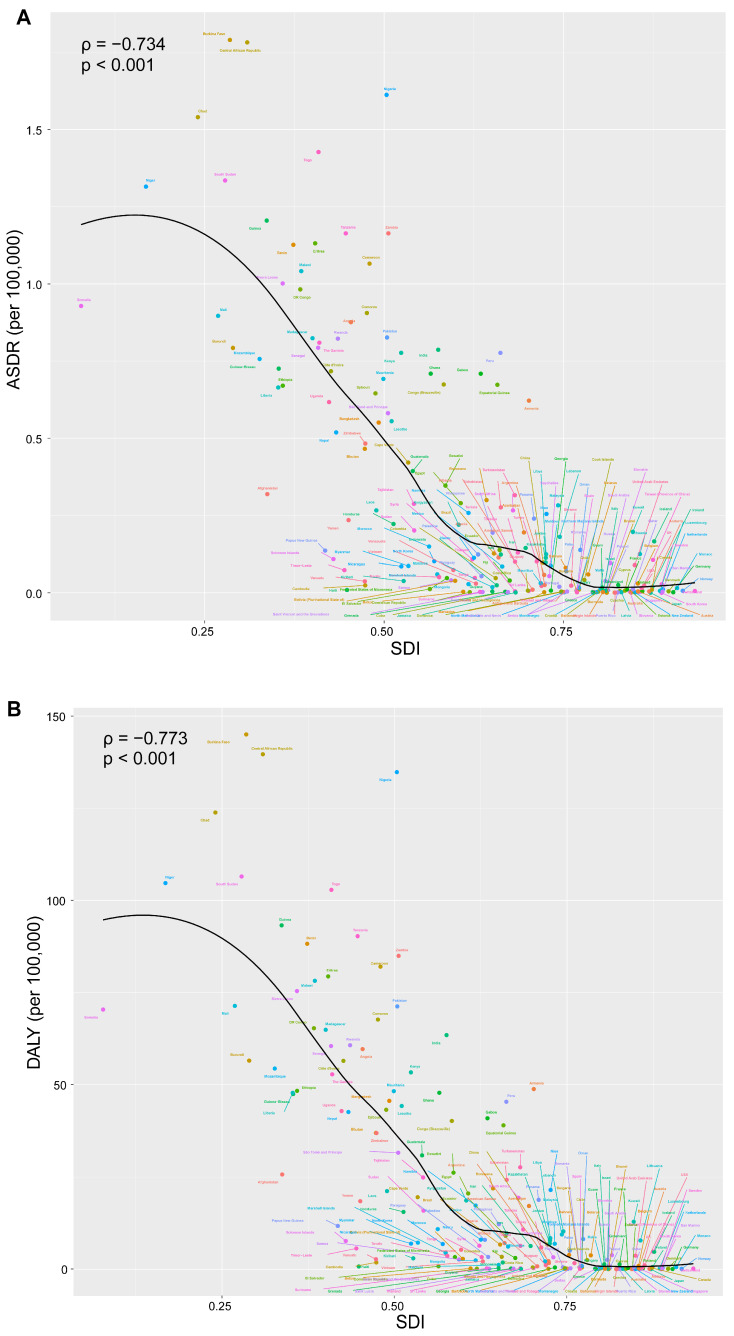
Correlation analyses of changes in incidence rate and SDI from 1990 to 2021. (**A**) Changes and correlation between ASDR and SDI from 1990 to 2021 in 204 countries. (**B**) Changes and correlation between age-standardized DALY rate and SDI from 1990 to 2021 in 204 countries. SDI: Socio-demographic Index.

At the national level, India recorded the highest number of deaths and DALYs in 2021, with 8294 (95%CI: 3079–15,045) and 674,441 (95%CI: 251,584–1,245,583) respectively (Tables S1 and S2). Notably, Burkina Faso exhibited the highest ASDR of 1.79 (95%CI: 1.04–2.68). And Guinea had the highest age-standardized DALY rate of 93.23 (95%CI: 53.17–141.74) (Figure 1A,B). From 1990 to 2021, Kuwait demonstrated the highest increase in ASDR, with an EAPC of 1.80 (95%CI: 0.51–3.10), while Georgia experienced the most significant decrease, with an EAPC of −9.17 (95%CI: −11.57 to −6.70). Kuwait exhibited the largest increase in age-standardized DALY rates, with an EAPC of 1.53 (95%CI: 0.21–2.87), whereas China had the largest decrease, with an EAPC of −8.27 (95%CI: −9.10 to −7.43) (Figure 2A,B). Additionally, correlation analyses revealed that as national and regional SDI increased, both the ASDR and age-standardized DALY rate tended to decrease. Specifically, the correlation coefficient for ASDR was ρ = −0.734 (*p* < 0.001), and for age-standardized DALY rates, it was ρ = −0.773 (*p* < 0.001) (Figure 3A,B).

### 3.2. Sex and Age Distribution of Deaths and DALYs

Mortality and DALY rates generally increase with age in both men and women, reflecting the growing prevalence of chronic and geriatric diseases, which leads to a higher number of healthy life years lost. At younger ages, men typically experience higher rates of mortality and DALYs compared to women. This discrepancy may be attributed to biological differences, sociocultural factors, and other influences. However, this gender gap tends to diminish with age.

**Figure 4:**
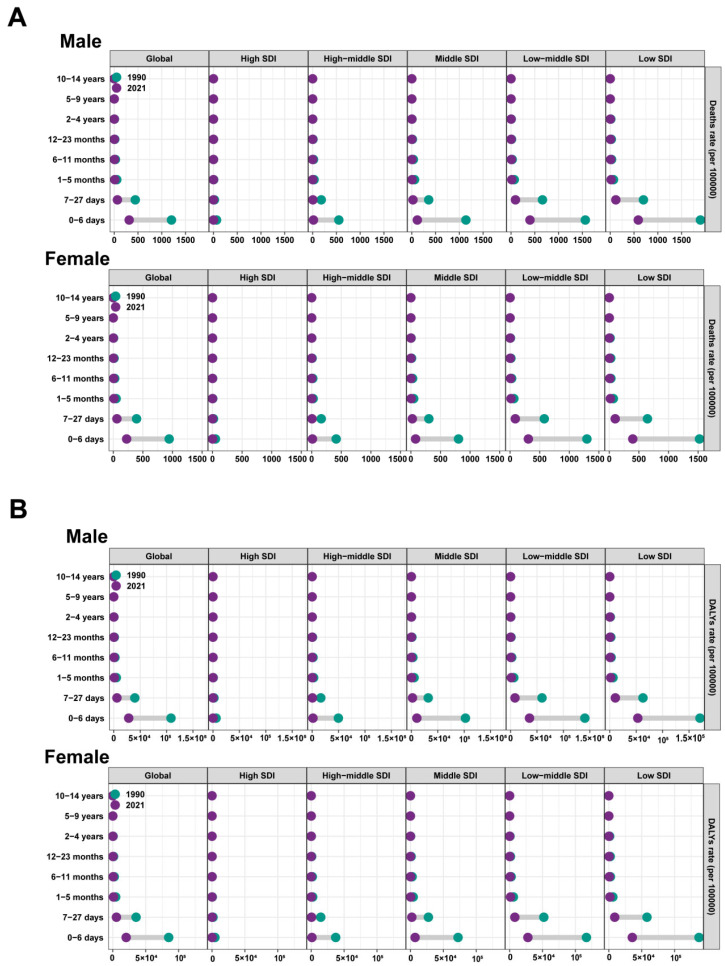
Changes in age-standardized rates of RSV-related LRI among different SDI quintiles and sex: (**A**) ASDR from 1990 to 2021; (**B**) Age-standardized DALY rate from 1990 to 2021. RSV: respiratory syncytial virus; LRI: lower respiratory infections; ASDR: age-standardized death rate; DALYs: disability-adjusted life years; SDI, Socio-demographic Index.

During the same period, the ASDR and age-standardized DALY rates for males were highest in the Low SDI quintile and lowest in the High SDI quintile. A similar pattern was observed for females. In addition, the trend from 1990 to 2021 was comparable for both genders. The ASDR and age-standardized DALY rates for females decreased across all five quintiles (Figure 4).

**Figure 5:**
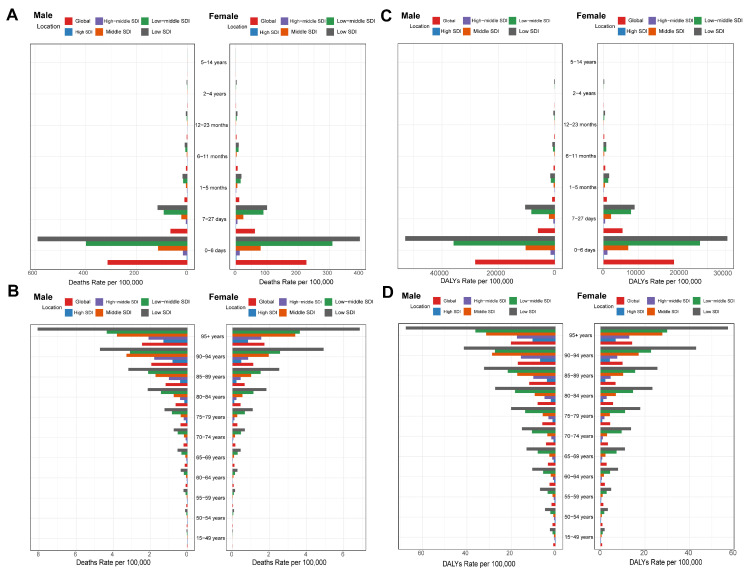
Death and DALY rates of RSV-related LRI of 19 age groups in different SDI quintiles and sex: (**A**) Death rate of RSV-related LRI of pediatric groups in different SDI quintiles; (**B**) Death rate of RSV-related LRI of adult groups in different SDI quintiles; (**C**) DALY rate of RSV-related LRI of pediatric groups in different SDI quintiles; (**D**) DALY rate of RSV-related LRI of adult groups in different SDI quintiles. The 11 age groups included adult groups (ages 15–49, 50–54, 55–59, 60–64, 65–69, 70–74, 75–79, 80–84, 85–89, 90–94, and 95+ years) and pediatric groups (ages 0–6 days, 7–27 days, 1–5 months, 6–11 months, 12–23 months, 2–4 years, 5–9 years, and 10–14 years); SDI: Socio-demographic Index. The influential factors for EAPC.

We divided the LRI patients into two groups—the adult group (15–49, 50–54, 55–59, 60–64, 65–69, 70–74, 75–79, 80–84, 85–89, 90–94, and 95+ years) and the pediatric group (0–6 days, 7–27 days, 1–5 months, 6–11 months, 12–23 months, 2–4, 5–9, and 10–14 years). In the adult group, there was a gradual increase in mortality with age, especially in the low SDI areas, with the highest mortality rates in the 95+ age group. In the children’s group, mortality was highest among children aged 0–6 days in low SDI areas. Global age-standardized DALY rates declined for both males and females in the child group, with the most pronounced declines in the low SDI regions. In contrast, in the adult group, global DALY rates increased for both males and females, with the most pronounced increases in the low SDI regions (Figure 5).

**Figure 6:**
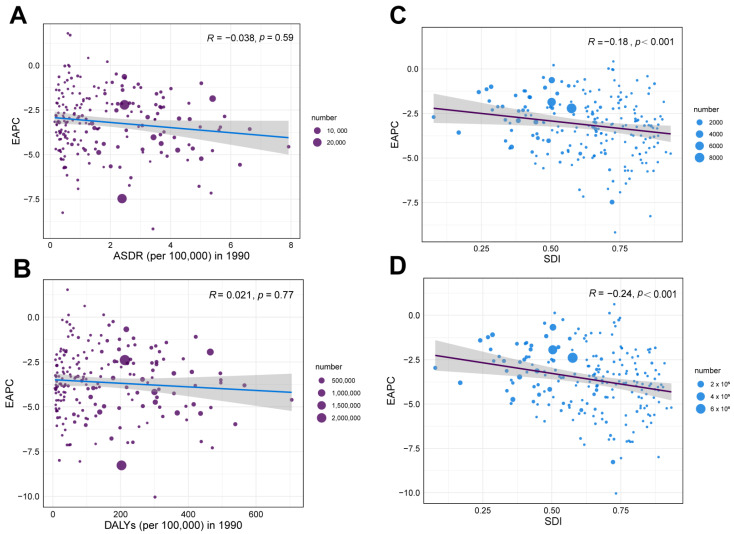
Correlation between EAPCs and age-standardized rate in 1990, and SDI in 2021: Correlation between EAPCs and ASDR in 1990 (**A**), and SDI in 2021 (**C**); The correlation between EAPCs and rate in 1990 (**B**), and SDI in 2021 (**D**). The circles represent 204 countries or territories, and the size of the circle represents the number of RSV-related LRI patients ρ: Pearson’s correlation coefficient; The grey part represents the confidence interval; RSV: respiratory syncytial virus; LRIs: lower respiratory infections; ASDR: age-standardized death rate; DALYs: disability-adjusted life years; SDI: Socio-demographic Index; EAPCs: estimated annual percentage changes.

We assessed the correlation coefficients between the 1990 EAPC, ASR, and the 2021 SDI. Our analysis revealed no significant correlation between ASDR (ρ = −0.038, *p* = 0.59) and age-standardized DALY rate (ρ = 0.021, *p* = 0.77), with the corresponding EAPC in 1990 (Figure 6A,B). In contrast, SDI showed negative correlations with both the ASDR (ρ = −0.18, *p* < 0.001) and the age-standardized DALY rate (ρ = −0.24, *p* < 0.001) concerning the corresponding EAPC in 2021 (Figure 6C,D). These results suggest that, in 2021, countries with higher SDI may have experienced a faster decline in ASR.

### 3.3. ARIMA Models Predict Trends in Burden of RSV-Related LRI

**Figure 7:**
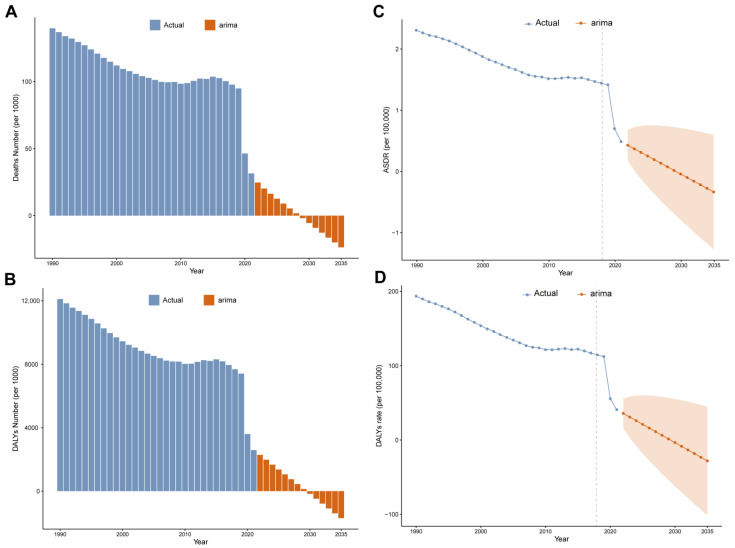
ARIMA models predict trends in disease burden of RSV-related LRI: (**A**) ARIMA model predicts the number of deaths; (**B**) ARIMA model predicts the number of DALYs; (**C**) ARIMA model predicts the ASDR rate; (**D**) ARIMA model predicts the age-standardized DALY rate. The orange part represents the confidence interval of the predicted value; RSV: respiratory syncytial virus; LRIs: lower respiratory infections; ASDR: age-standardized death rate; DALYs: disability-adjusted life years.

From 1990 to 2020, the global mortality rate decreased significantly, from over 100 deaths per 1000 people to approximately 50 deaths per 1000 people, reflecting improvements in sanitation and advancements in medical technology. The ARIMA model predicts that mortality will continue to decline from 2020 to 2035, albeit at a progressively slower rate, with a further reduction in the number of deaths projected for 2035, highlighting the long-term benefits of public health interventions. Additionally, the model predicts that the decline in mortality will persist in 2035, although at a slower pace (Figure 7A).

During the same period, the global number of DALYs decreased from nearly 12,000 per 1000 people to about 2000, reflecting a reduced disease burden due to public health strategies and improved healthcare quality. The ARIMA model predicts that the number of DALYs will continue to decline—albeit at a slower rate—from 2020 to 2035, with an even lower level expected by 2035 (Figure 7B). This underscores the importance of continued investment in health promotion and disease prevention.

From 1990 to 2020, the global ASDR decreased significantly, from over 2.0 deaths per 100,000 people to approximately 0.5 deaths per 100,000 people. The ARIMA model predicts that this downward trend in ASDR will continue from 2020 to 2035, although at a slower rate. Similarly, the rate of DALYs decreased from nearly 200 cases per 100,000 people to about 50 cases per 100,000 people, with projections indicating further declines from 2020 to 2035, reaching an even lower level by 2035 (Figure 7C,D).

## 4. Discussion

In this study, we systematically analyzed the current burden and temporal trends of lower respiratory infections due to respiratory syncytial virus by global region, age, sex, geographic location, and socioeconomic status using GBD data from 1990 to 2021. Our findings reveal a significant decline in the worldwide burden of LRI due to RSV, consistent with the previous reports [14], reflecting a marked reduction at the global level. These results suggest that human efforts to combat LRIs have led to more favorable outcomes.

Over the past few decades, significant progress has been made in all aspects of RSV diagnosis, treatment, and prevention. In the early days, RSV diagnosis relied on viral culture and immunofluorescence methods, which were time-consuming and placed significant demands on the laboratory environment. However, in recent years, the popularity of molecular diagnostic techniques, especially real-time reverse transcription polymerase chain reaction (RT-PCR) and antigen detection, has not only improved the speed of diagnosis but also significantly enhanced sensitivity and specificity, enabling the accurate identification of RSV infection at an early stage [15,16]. These advances have improved our ability to monitor and track RSV outbreaks, which is epidemiologically important for public health surveillance and intervention strategies. Parallel to these diagnostic improvements, the last three decades have seen a transformation in RSV therapeutics and prophylactic strategies, contributing to a notable reduction in the global burden of RSV-related morbidity and mortality. Palivizumab, an anti-F protein mAb, reduced RSV hospitalization rates by 51% in high-risk infants through passive immunization, with meta-analyses confirming its role in lowering ICU admissions and healthcare costs [17]. Small-molecule antivirals targeting RSV fusion (F) proteins—such as benzimidazole derivatives and GS-5806—emerged as promising alternatives. These agents disrupt viral entry by stabilizing the F protein’s prefusion conformation, showing efficacy in reducing viral replication in clinical trials [18,19]. Vaccine development, though historically hindered by the legacy of the failed formalin-inactivated vaccine, gained momentum with prefusion-stabilized F protein (preF) designs. By 2021, over 15 candidates—including subunit and mRNA vaccines—were undergoing clinical trials, targeting infants, pregnant women, and the elderly [17,20]. Ongoing advances in diagnostic methods, treatment strategies, and prophylactic interventions plays a crucial role in alleviating the burden of RSV-induced lower respiratory infections.

Despite the global decline in the burden of RSV-associated LRIs, there are still significant differences between countries and regions. The SDI is a comprehensive indicator of a country’s socio-economic status and is closely related to factors such as education level, per capita income, and fertility rate. In this study, we found a higher RSV-related LRI burden in low SDI regions. The sub-Saharan region of West Africa had the highest number of RSV-related deaths globally, while South Asia reported the highest number of DALYs. At the same time, both mortality and DALYs were higher in low SDI regions than in high SDI regions, and the rate of reduction in RSV burden was relatively slow in these regions. Several studies have shown that public health systems in low SDI countries and regions face greater challenges in responding to respiratory infectious diseases [21,22,23]. In contrast, high SDI regions (e.g., high-income countries) have significantly lower RSV burdens due to stronger medical facilities, health awareness, and effective public health policies. The number of LRI deaths and DALYs in high-income Asia-Pacific regions was significantly lower than in other regions, and the reduction in mortality and DALYs was greater. Previous studies have also noted that high-income countries can significantly reduce the LRI burden through better health systems and vaccination strategies [24]. In summary, the level of economic and social development plays a crucial role in addressing the RSV-associated LRI burden. In the field of global health, policymakers and the international community need to pay more attention to building public health in low-income and lower-middle-income countries to reduce such regional disparities and support the achievement of global health goals. At the same time, there is a need to develop more effective vaccines and scale up interventions. Countries that have not yet adopted the RSV vaccine need to be encouraged to do so aggressively to reduce the burden of RSV in these regions.

The results of our study suggest that children and the elderly bear a disproportionate burden of lower respiratory infections attributable to RSV. Children—particularly infants under six months of age—are especially vulnerable to RSV due to the incomplete development of their immune systems, which results in higher rates of hospitalization and mortality. Infant mortality is particularly pronounced in low SDI areas, where limited medical resources and relatively low levels of public health infrastructure exacerbate the impact of RSV [12]. Early prophylaxis is crucial in this population, and monoclonal antibodies have been shown to significantly reduce the incidence of severe illness and hospitalization caused by RSV. However, the feasibility and effectiveness of vaccination still need to be improved [25]. Strengthening maternal and child health interventions, particularly by providing RSV immunoprophylaxis to pregnant women in low SDI areas, can also significantly reduce the risk of infection in infants [26]. Furthermore, the burden of RSV is notably high in the elderly population, especially those with chronic diseases. These individuals are more susceptible to severe lower respiratory tract infections due to age-related declines in immune function and poorer overall health. Other studies have similarly emphasized that the elderly, particularly those over 70 years of age, are a high-risk group for RSV-induced respiratory infections [7]. To mitigate this burden, more targeted prevention and management strategies are essential for this demographic [27].

Our findings should be interpreted in the context of the unprecedented disruptions caused by the COVID-19 pandemic. While our study period extends to 2021, the implementation of non-pharmaceutical interventions (NPIs) during 2020–2022 significantly altered RSV epidemiology through the following three key mechanisms: (1) Temporal shift: Global NPIs initially suppressed RSV transmission by 40–80% in 2020, followed by off-season surges upon NPI relaxation, particularly in 2021 [28]. (2) Age redistribution: Reduced viral exposure in infants during lockdowns created an “immunity gap”, leading to elevated hospitalization rates among 1–2-year-olds in subsequent seasons [29]. (3) Diagnostic interference: Co-circulation with SARS-CoV-2 and overlapping clinical presentations may have affected case ascertainment in 2021 [30]. These pandemic-related anomalies were excluded from our trend projections but highlight the complex interplay between population immunity and respiratory virus dynamics.

Overall, global mortality from RSV-induced lower respiratory infections follows a complex pattern, influenced by multiple factors such as demographic characteristics, economic development, and the quality of healthcare. The burden of RSV-induced LRIs is particularly pronounced in regions with low SDI, as well as among neonates and the elderly. Therefore, it is crucial to develop targeted prevention and control strategies tailored to different SDI regions and population groups to mitigate the global impact of RSV-induced LRIs.

To the best of our knowledge, this study is the most recent to examine the global, regional, and national burden of RSV. However, there are several limitations to consider. First, the analysis is based on data from the GBD 2021 study, which only covers up to 2021. We lack data for 2022–2024 due to the ongoing updates to the GBD database. Future research will hopefully include these years to provide a more comprehensive view. Second, the data primarily focus on global, regional, and national levels, with limited information at the individual level. As a result, we were unable to conduct a more detailed analysis of the burden of RSV at the individual level. Third, while the burden of RSV is influenced by a variety of complex factors, we did not account for potential future changes in these factors when projecting the global burden of RSV, which could lead to biased projections. Subsequent studies should integrate NASA’s MERRA-2 climate datasets with Gavi’s vaccine deployment metrics through hybrid ARIMAX-LSTM architectures to enhance predictive granularity. Nevertheless, this study provides valuable insights to raise public awareness of the burden of RSV and offers data to support the development and implementation of relevant policies, interventions, and measures at the global, regional, and national levels.

## 5. Conclusions

This study reveals a declining trend in global mortality rates and DALYs due to RSV-induced lower respiratory infections from 1990 to 2021. However, the burden remains particularly pronounced in low SDI regions, as well as among newborns and the elderly. These findings underscore the significant disparities in the burden of RSV-induced LRIs across regions with varying levels of socioeconomic development. In light of these findings, there is an urgent need to develop and implement effective prevention strategies tailored to the specific needs of different regions and populations. Key measures should focus on improving access to screening and treatment services in less developed regions, implementing targeted prevention strategies, and addressing modifiable risk factors. Future research should prioritize the development of cost-effective interventions that are adaptable to local environments and resources, with the aim of reducing mortality from RSV-induced LRIs. Additionally, efforts should be made to enhance data collection and improve data quality in resource-poor areas. This would enable more accurate monitoring of trends in RSV-induced LRIs and provide a clearer assessment of the effectiveness of existing and future interventions.

## Figures and Tables

**Table 1:** Death cases and ASDRs of RSV-related LRI in 1990 and 2021, with temporal trends from 1990 to 2021.

	Deaths Cases (95% CI)		ASDR (95% CI)		1990–2021 EAPCs (95%CI)
	1990	2021	1990	2021	
**Global**	139,762 (123,666–158,110)	31,525 (23,348–41,871)	2.31 (2.05–2.60)	0.49 (0.36–0.65)	−2.62 (−3.33 to −1.91)
**SDI**					
High SDI	3391 (3102–3628)	290 (211–396)	0.38 (0.35–0.41)	0.02 (0.01–0.03)	−3.19 (−4.84 to −1.51)
High-middle SDI	10,300 (9268–11,698)	626 (467–841)	1.16 (1.05–1.32)	0.06 (0.05–0.08)	−5.24 (−6.30 to −4.16)
Middle SDI	40,684 (36,610–45,407)	3563 (2585–4721)	2.13 (1.93–2.37)	0.21 (0.15–0.28)	−4.10 (−4.98 to −3.21)
Low-middle SDI	49,350 (42,974–56,450)	11,772 (7763–16,625)	2.84 (2.49–3.25)	0.66 (0.43–0.93)	−2.38 (−3.10 to −1.64)
Low SDI	35,948 (29,715–42,705)	15,264 (10,766–20,495)	3.85 (3.20–4.55)	1.01 (0.72–1.34)	−2.39 (−2.99 to −1.79)
**Central Europe, eastern Europe, and central Asia**					
Central Asia	4344 (4034–4707)	222 (118–365)	4.64 (4.31–5.02)	0.23 (0.12–0.38)	−3.98 (−5.37 to −2.57)
Central Europe	841 (795–883)	9 (2–22)	0.92 (0.87–0.96)	0.01 (0.00–0.01)	−4.54 (−7.04 to −1.98)
Eastern Europe	1073 (1017–1134)	68 (29–130)	0.68 (0.64–0.72)	0.04 (0.02–0.07)	−4.18 (−5.48 to −2.86)
**High income region**					
High-income Asia Pacific	757 (681–817)	2 (0–12)	0.50 (0.45–0.54)	0.00 (0.00–0.00)	−5.77 (−9.62 to −1.75)
High-income North America	967 (870–1042)	113 (55–205)	0.30 (0.27–0.32)	0.02 (0.01–0.04)	−3.23 (−4.60 to −1.85)
Western Europe	1292 (1159–1399)	97 (62–143)	0.27 (0.25–0.29)	0.01 (0.01–0.01)	−3.16 (−5.10 to −1.17)
Australasia	42 (39–46)	0 (0–1)	0.23 (0.22–0.25)	0.00 (0.00–0.00)	−5.04 (−8.35 to −1.61)
**Latin America and Caribbean**					
Andean Latin America	1404 (1225–1590)	284 (106–464)	2.77 (2.43–3.12)	0.48 (0.18–0.79)	−3.12 (−4.26 to −1.96)
Caribbean	613 (516–733)	2 (0–11)	1.54 (1.31–1.82)	0.01 (0.00–0.03)	−3.75 (−6.99 to −0.39)
Southern Latin America	277 (262–291)	32 (13–61)	0.59 (0.55–0.62)	0.04 (0.02–0.08)	−1.51 (−3.69–0.72)
Tropical Latin America	2064 (1841–2295)	263 (78–586)	1.37 (1.23–1.51)	0.12 (0.04–0.28)	−3.22 (−4.53 to −1.89)
Central Latin America	2779 (2571–3032)	212 (142–305)	1.29 (1.20–1.39)	0.10 (0.07–0.15)	−2.46 (−3.82 to −1.08)
**North Africa and Middle East**					
North Africa and Middle East	10,774 (9198–13,390)	1039 (560–1796)	2.17 (1.85–2.67)	0.20 (0.11–0.34)	−3.86 (−4.46 to −3.25)
**South Asia**					
South Asia	41,103 (34,240–48,132)	11,561 (5798–18,693)	2.64 (2.21–3.08)	0.77 (0.39–1.25)	−2.20 (−2.69 to −1.71)
**Southeast Asia, east Asia, and Oceania**					
East Asia	25,950 (22,337–30,046)	908 (513–1516)	2.34 (2.02–2.70)	0.10 (0.06–0.16)	−7.31 (−8.16 to −6.45)
Oceania	305 (247–380)	23 (11–43)	3.05 (2.50–3.76)	0.12 (0.06–0.22)	−2.00 (−4.03–0.07)
Southeast Asia	11,749 (10,245–13,843)	398 (259–584)	2.09 (1.83–2.44)	0.07 (0.05–0.11)	−3.93 (−5.53 to −2.31)
**Sub-Saharan Africa**					
Central Sub-Saharan Africa	3460 (2530–4456)	1537 (729–2437)	3.31 (2.48–4.17)	0.98 (0.48–1.50)	−3.06 (−3.36 to −2.77)
Eastern Sub-Saharan Africa	11,960 (9743–14,547)	4505 (2917–6559)	3.31 (2.73–3.96)	0.85 (0.57–1.23)	−2.79 (−3.45 to −2.13)
Southern Sub-Saharan Africa	1479 (1295–1693)	245 (98–490)	2.15 (1.90–2.44)	0.33 (0.13–0.67)	−1.50 (−3.34–0.37)
Western Sub-Saharan Africa	16,519 (13,585–19,646)	9996 (5877–14,968)	4.47 (3.68–5.30)	1.34 (0.80–2.00)	−1.80 (−2.41 to −1.20)

**Table 2:** DALYs and age-standardized DALY rates of RSV-related LRIs in 1990 and 2021, with temporal trends from 1990 to 2021.

	DALYs (95% CI)		Age-Standardized DALY Rate (95% CI)		1990–2021 EAPCs (95%CI)
	1990	2021	1990	2021	
**Global**	12,105,847 (10,665,949–13,740,784)	2,591,507 (1,902,003–3,468,792)	193.49 (170.61–219.46)	40.82 (29.91–54.59)	−2.77 (−3.44 to −2.09)
**SDI**					
High SDI	122,762 (115,754–130,626)	7790 (5622–10,838)	17.16 (16.12–18.35)	0.89 (0.63–1.23)	−3.38 (−4.85 to −1.88)
High-middle SDI	866,214 (774,684–990,312)	28,413 (21,346–37,666)	96.90 (86.68–110.78)	3.84 (2.85–5.13)	−6.34 (−7.34 to −5.33)
Middle SDI	3,563,942 (3,200,439–3,987,783)	251,513 (179,101–340,161)	180.15 (161.94–201.38)	15.34 (10.88–20.86)	−4.54 (−5.41 to −3.67)
Low-middle SDI	4,368,503 (3,799,151–5,000,857)	982,202 (651,937–1,386,106)	244.40 (212.81–279.98)	52.79 (35.00–74.55)	−2.58 (−3.31 to −1.84)
Low SDI	3,177,072 (2,621,925–3,775,665)	1,321,025 (932,144–1,775,047)	326.85 (269.73–389.51)	80.35 (56.66–107.90)	−2.56 (−3.17 to −1.95)
**Central Europe, eastern Europe, and central Asia**					
Central Asia	386,572 (358,853–418,955)	19,064 (9879–31,308)	410.33 (381.01–444.66)	19.29 (10.02–31.67)	−4.10 (−5.50 to −2.68)
Central Europe	64,501 (60,872–67,975)	302 (102–665)	73.73 (69.47–77.82)	0.36 (0.13–0.76)	−5.54 (−8.07 to −2.94)
Eastern Europe	89,677 (84,819–95,109)	3395 (1479–6437)	58.39 (55.12–61.98)	2.55 (1.09–4.89)	−5.07 (−6.33 to −3.79)
**High income region**					
High-income Asia Pacific	21,608 (19,934–23,193)	32 (3–167)	16.94 (15.45–18.46)	0.01 (0.00–0.06)	−6.13 (−9.95 to −2.15)
High-income North America	31,180 (29,641–32,616)	3149 (1488–5682)	11.83 (11.28–12.35)	0.96 (0.45–1.74)	−2.74 (−4.14 to −1.33)
Western Europe	33,033 (31,404–34,542)	1547 (974–2258)	10.08 (9.71–10.45)	0.28 (0.18–0.41)	−3.64 (−5.55 to −1.69)
Australasia	1841 (1718–1974)	11 (2–25)	11.12 (10.34–11.99)	0.04 (0.01–0.09)	−5.31 (−8.68 to −1.81)
**Latin America and Caribbean**					
Andean Latin America	121,704 (106,006–138,172)	16,774 (6358–28,740)	225.47 (196.65–255.68)	27.78 (10.53–47.55)	−3.82 (−5.01 to −2.61)
Caribbean	51,753 (43,055–62,437)	161 (46–808)	123.00 (102.62–147.91)	0.41 (0.12–2.06)	−3.94 (−7.17 to −0.59)
Southern Latin America	19,127 (18,027–20,171)	919 (398–1779)	38.14 (35.97–40.18)	1.67 (0.74–3.26)	−3.19 (−5.33 to −1.00)
Tropical Latin America	175,597 (156,002–196,127)	12,370 (3599–27,803)	109.81 (97.68–122.46)	6.58 (1.93–14.88)	−4.42 (−5.80 to −3.01)
Central Latin America	240,956 (222,737–263,718)	15,563 (10,261–22,529)	104.04 (96.20–113.67)	7.93 (5.23–11.48)	−2.68 (−4.05 to −1.30)
**North Africa and Middle East**					
North Africa and Middle East	953,149 (813,177–1,186,437)	79,031 (40,379–138,867)	185.69 (158.49–230.93)	13.74 (7.08–24.08)	−4.37 (−5.00 to −3.74)
**South Asia**					
South Asia	3,645,872 (3,031,824–4,273,297)	959,423 (484,294–1,553,206)	228.41 (190.32–267.49)	63.00 (31.79–102.03)	−2.36 (−2.86 to −1.85)
**Southeast Asia, east Asia, and Oceania**					
East Asia	2,268,332 (1,947,322–2,627,318)	45,581 (25,630–76,212)	198.61 (170.68–229.97)	6.68 (3.69–11.12)	−8.14 (−8.98 to −7.30)
Oceania	27,062 (21,928–33,795)	2105 (1010–3884)	259.24 (210.30–322.38)	10.51 (5.05–19.34)	−1.99 (−4.02–0.09)
Southeast Asia	1,030,723 (898,445–1,218,338)	27,229 (17,728–40,560)	177.34 (154.64–209.25)	4.89 (3.20–7.28)	−4.56 (−6.18 to −2.91)
**Sub-Saharan Africa**					
Central Sub-Saharan Africa	305,071 (222,276–393,719)	128,479 (59,859–204,950)	272.16 (199.13–350.26)	65.72 (31.33–103.94)	−3.62 (−3.96 to −3.28)
Eastern Sub-Saharan Africa	1,053,436 (856,319–1,285,137)	384,101 (246,486–559,266)	272.46 (221.83–331.61)	62.48 (40.51–90.90)	−3.08 (−3.76 to −2.40)
Southern Sub-Saharan Africa	127,066 (110,619–146,145)	19,442 (7745–39,009)	172.02 (150.42–197.12)	24.96 (9.96–50.12)	−1.68 (−3.51–0.18)
Western Sub-Saharan Africa	1,457,578 (1,198,455–1,733,011)	872,819 (513,360–1,305,845)	379.38 (309.14–452.76)	108.55 (63.85–162.60)	−1.94 (−2.55 to −1.32)

## Data Availability

The data used in this study are available free of charge online at https://vizhub.healthdata.org/gbd-results (accessed on 20 December 2024) on request. The datasets used and/or analyzed during the current study are available from the corresponding author upon reasonable request.

## Supplementary Materials

The following supporting information can be downloaded at: https://www.mdpi.com/article/10.3390/tropicalmed10080223/s1, Table S1: The death cases and ASDR of RSV-related LRI in 1990 and 2021, with Temporal Trends from 1990 to 2021 in 204 countries or territories.; Table S2: The death cases and DALY of RSV-related LRI in 1990 and 2021, with Temporal Trends from 1990 to 2021 in 204 countries or territories.

## References

[1] Hall C.B., Weinberg G.A., Iwane M.K., Blumkin A.K., Edwards K.M., Staat M.A., Auinger P., Griffin M.R., Poehling K.A., Erdman D. (2009). The burden of respiratory syncytial virus infection in young children. N. Engl. J. Med..

[2] Nair H., Nokes D.J., Gessner B.D., Dherani M., Madhi S.A., Singleton R.J., O’Brien K.L., Roca A., Wright P.F., Bruce N. (2010). Global burden of acute lower respiratory infections due to respiratory syncytial virus in young children: A systematic review and meta-analysis. Lancet.

[3] Raffaldi I., Castagno E. (2024). The Epidemiology of Respiratory Syncytial Virus: New Trends and Future Perspectives. Viruses.

[4] Shi T., McAllister D.A., O’Brien K.L., Simoes E.A.F., Madhi S.A., Gessner B.D., Polack F.P., Balsells E., Acacio S., Aguayo C. (2017). Global, regional, and national disease burden estimates of acute lower respiratory infections due to respiratory syncytial virus in young children in 2015: A systematic review and modelling study. Lancet.

[5] Alfano F., Bigoni T., Caggiano F.P., Papi A. (2024). Respiratory Syncytial Virus Infection in Older Adults: An Update. Drugs Aging.

[6] Karron R.A., Singleton R.J., Bulkow L., Parkinson A., Kruse D., DeSmet I., Indorf C., Petersen K.M., Leombruno D., Hurlburt D. (1999). Severe respiratory syncytial virus disease in Alaska native children. RSV Alaska Study Group. J. Infect. Dis..

[7] Du Y., Yan R., Wu X., Zhang X., Chen C., Jiang D., Yang M., Cao K., Chen M., You Y. (2023). Global burden and trends of respiratory syncytial virus infection across different age groups from 1990 to 2019: A systematic analysis of the Global Burden of Disease 2019 Study. Int. J. Infect. Dis..

[8] Díez-Domingo J., Pérez-Yarza E.G., Melero J.A., Sánchez-Luna M., Aguilar M.D., Blasco A.J., Alfaro N., Lázaro P. (2014). Social, economic, and health impact of the respiratory syncytial virus: A systematic search. BMC Infect. Dis..

[9] GBD 2019 Diseases and Injuries Collaborators (2020). Global burden of 369 diseases and injuries in 204 countries and territories, 1990–2019: A systematic analysis for the Global Burden of Disease Study 2019. Lancet.

[10] Divarathne M.V.M., Ahamed R.R., Noordeen F. (2019). The Impact of RSV-Associated Respiratory Disease on Children in Asia. J. Pediatr. Infect. Dis..

[11] Thompson W.W., Shay D.K., Weintraub E., Brammer L., Bridges C.B., Cox N.J., Fukuda K. (2004). Influenza-associated hospitalizations in the United States. JAMA.

[12] Li Y., Wang X., Blau D.M., Caballero M.T., Feikin D.R., Gill C.J., Madhi S.A., Omer S.B., Simões E.A.F., Campbell H. (2022). Global, regional, and national disease burden estimates of acute lower respiratory infections due to respiratory syncytial virus in children younger than 5 years in 2019: A systematic analysis. Lancet.

[13] Global Burden of Disease Cancer Collaboration (2017). Global, regional, and national cancer incidence, mortality, years of life lost, years lived with disability, and disability-adjusted life-years for 32 cancer groups, 1990 to 2015: A systematic analysis for the global burden of disease study. JAMA Oncol..

[14] Wu M., Wu Q., Liu D., Zu W., Zhang D., Chen L. (2024). The global burden of lower respiratory infections attributable to respiratory syncytial virus in 204 countries and territories, 1990–2019: Findings from the Global Burden of Disease Study 2019. Intern. Emerg. Med..

[15] Chartrand C., Tremblay N., Renaud C., Papenburg J. (2015). Diagnostic Accuracy of Rapid Antigen Detection Tests for Respiratory Syncytial Virus Infection: Systematic Review and Meta-analysis. J. Clin. Microbiol..

[16] Ferrani S., Prazuck T., Béchet S., Lesne F., Cohen R., Levy C. (2023). Diagnostic accuracy of a rapid antigen triple test (SARS-CoV-2, respiratory syncytial virus, and influenza) using anterior nasal swabs versus multiplex RT-PCR in children in an emergency department. Infect. Dis. Now..

[17] El-Atawi K., De Luca D., Ramanathan R., Luna M.S., Alsaedi S., Wahab M.G.A., Hamdi M., Saleh M. (2023). Efficacy and Safety of Palivizumab as a Prophylaxis for Respiratory Syncytial Virus (RSV) Disease: An Updated Systemic Review and Meta-Analysis. Cureus.

[18] Cichero E., Calautti A., Francesconi V., Tonelli M., Schenone S., Fossa P. (2021). Probing In Silico the Benzimidazole Privileged Scaffold for the Development of Drug-like Anti-RSV Agents. Pharmaceuticals.

[19] Cox R., Plemper R.K. (2016). Structure-guided design of small-molecule therapeutics against RSV disease. Expert. Opin. Drug Discov..

[20] Chirikov V., Botteman M., Simões E.A.F. (2022). The Long-Term Healthcare Utilization and Economic Burden of RSV Infection in Children ≤5 Years in Japan: Propensity Score Matched Cohort Study. Clin. Outcomes Res..

[21] Clark J., Kochovska S., Currow D.C. (2022). Burden of respiratory problems in low-income and middle-income countries. Curr. Opin. Support. Palliat. Care.

[22] GBD 2019 Chronic Respiratory Diseases Collaborators (2023). Global burden of chronic respiratory diseases and risk factors, 1990–2019: An update from the Global Burden of Disease Study 2019. eClinicalMedicine.

[23] Meghji J., Mortimer K., Agusti A., Allwood B.W., Asher I., Bateman E.D., Bissell K., Bolton C.E., Bush A., Celli B. (2021). Improving lung health in low-income and middle-income countries: From challenges to solutions. Lancet.

[24] Du Z., Pandey A., Moghadas S.M., Bai Y., Wang L., Matrajt L., Singer B.H., Galvani A.P. (2025). Impact of RSVpreF vaccination on reducing the burden of respiratory syncytial virus in infants and older adults. Nat. Med..

[25] Esposito S., Raya B.A., Baraldi E., Flanagan K., Torres F.M., Tsolia M., Zielen S. (2022). RSV Prevention in All Infants: Which Is the Most Preferable Strategy?. Front. Immunol..

[26] Gong X., Luo E., Fan L., Zhang W., Yang Y., Du Y., Yang X., Xing S. (2023). Clinical research on RSV prevention in children and pregnant women: Progress and perspectives. Front. Immunol..

[27] Shi T., Denouel A., Tietjen A.K., Campbell I., Moran E., Li X., Campbell H., Demont C., Nyawanda B.O., Chu H. (2020). Global Disease Burden Estimates of Respiratory Syncytial Virus-Associated Acute Respiratory Infection in Older Adults in 2015: A Systematic Review and Meta-Analysis. J. Infect. Dis..

[28] Bents S.J., Viboud C., Grenfell B.T., Hogan A.B., Tempia S., von Gottberg A., Moyes J., Walaza S., Hansen C., Cohen C. (2023). Modeling the impact of COVID-19 nonpharmaceutical interventions on respiratory syncytial virus transmission in South Africa. Influenza Other Respir. Viruses.

[29] Al-Jwadi R.F., Mills E.H.A., Torp-Pedersen C., Andersen M.P., Jørgensen I.M. (2023). Consequences of COVID-19-related lockdowns and reopenings on emergency hospitalizations in pediatric patients in Denmark during 2020-2021. Eur. J. Pediatr..

[30] Angelova A., Atanasova M., Ketev K., Halil Z., Paskaleva I., Lengerova G., Dimcheva T., Korsun N., Murdjeva M. (2023). Severe SARS-CoV-2 and respiratory syncytial virus co-infection in two children. Folia Med..

