# Supplementary material for: Contribution of Respiratory Syncytial Virus to Burden of Lower Respiratory Tract Infections: A Global Analysis of 204 Countries and Territories, 1990–2021
Source: Trop Med Infect Dis. 2025 Aug 11;10(8):223. doi: 10.3390/tropicalmed10080223 (PMC12390104; doi:10.3390/tropicalmed10080223)
Supplement: Supplementary file 1 [file tropicalmed-10-00223-s001.zip › tropicalmed-3618460-supplementary.pdf]

**Table S1.** The death cases and ASDR of RSV-related LRI in 1990 and 2021, with Temporal Trends from 1990 to 2021 in 204 countries or territories.

| location | Num_1990 | ASDR_1990 | Num_2021 | ASDR_2021 | EAPC_ASDR |
|---|---|---|---|---|---|
| China | 25,508 (21,897 - 29,533) | 2.39 (2.06 - 2.75) | 857 (484 - 1,432) | 0.10 (0.06 - 0.16) | -7.47 (-8.31 to -6.63) |
| North Korea | 363 (275 - 473) | 1.53 (1.18 - 1.98) | 19 (10 - 33) | 0.10 (0.05 - 0.18) | -4.30 (-5.48 to -3.11) |
| Taiwan (Province of China) | 79 (75 - 84) | 0.57 (0.53 - 0.60) | 33 (18 - 54) | 0.11 (0.06 - 0.18) | -0.83 (-2.20 - 0.56) |
| Cambodia | 1,179 (986 - 1,448) | 6.30 (5.32 - 7.69) | 4 (1 - 8) | 0.02 (0.01 - 0.05) | -5.57 (-8.37 to -2.68) |
| Indonesia | 3,471 (2,883 - 4,260) | 1.61 (1.34 - 1.96) | 50 (19 - 106) | 0.02 (0.01 - 0.05) | -4.88 (-6.80 to -2.91) |
| Laos | 404 (307 - 510) | 5.39 (4.13 - 6.77) | 21 (9 - 39) | 0.27 (0.11 - 0.51) | -4.78 (-6.04 to -3.51) |
| Malaysia | 118 (98 - 138) | 0.66 (0.56 - 0.77) | 68 (30 - 135) | 0.28 (0.12 - 0.56) | 1.70 (0.62 - 2.80) |
| Maldives | 5 (4 - 6) | 1.27 (1.05 - 1.59) | 0 (0 - 0) | 0.02 (0.01 - 0.03) | -4.99 (-6.92 to -3.02) |
| Myanmar | 2,549 (1,966 - 3,197) | 5.02 (3.88 - 6.27) | 44 (21 - 76) | 0.09 (0.04 - 0.15) | -5.23 (-7.14 to -3.27) |
| Philippines | 1,893 (1,598 - 2,273) | 2.20 (1.90 - 2.60) | 181 (83 - 347) | 0.19 (0.09 - 0.37) | -2.52 (-3.89 to -1.14) |
| Sri Lanka | 71 (62 - 81) | 0.50 (0.44 - 0.56) | 1 (0 - 4) | 0.01 (0.00 - 0.02) | -4.03 (-6.61 to -1.37) |
| Thailand | 382 (299 - 484) | 0.80 (0.64 - 1.01) | 4 (1 - 11) | 0.01 (0.00 - 0.01) | -4.17 (-7.00 to -1.27) |
| Timor-Leste | 67 (55 - 84) | 4.57 (3.77 - 5.62) | 1 (1 - 2) | 0.07 (0.04 - 0.12) | -5.31 (-7.13 to -3.46) |
| Vietnam | 1,586 (1,305 - 1,975) | 1.78 (1.48 - 2.20) | 23 (7 - 53) | 0.03 (0.01 - 0.07) | -4.40 (-6.36 to -2.41) |
| Fiji | 6 (5 - 8) | 0.78 (0.64 - 0.96) | 0 (0 - 1) | 0.04 (0.02 - 0.07) | -1.17 (-3.42 - 1.13) |
| Kiribati | 1 (1 - 2) | 1.19 (0.92 - 1.49) | 0 (0 - 0) | 0.04 (0.02 - 0.07) | -3.23 (-5.09 to -1.35) |
| Marshall Islands | 1 (1 - 1) | 1.22 (0.99 - 1.45) | 0 (0 - 0) | 0.06 (0.03 - 0.10) | -1.84 (-3.96 - 0.33) |
| Federated | 2 (2 - 3) | 1.63 (1.28 - 1.98) | 0 (0 - 0) | 0.05 (0.02 - 0.08) | -3.51 (-5.45 to -1.53) |

| location | Num_1990 | ASDR_1990 | Num_2021 | ASDR_2021 | EAPC_ASDR |
|---|---|---|---|---|---|
| States of Micronesia | | | | | |
| Papua New Guinea | 251 (200 - 318) | 3.77 (3.05 - 4.74) | 21 (10 - 39) | 0.14 (0.06 - 0.25) | -2.33 (-4.36 to -0.27) |
| Samoa | 3 (2 - 4) | 1.30 (0.99 - 1.62) | 0 (0 - 0) | 0.04 (0.02 - 0.07) | -3.29 (-5.12 to -1.43) |
| Solomon Islands | 14 (11 - 18) | 2.70 (2.12 - 3.36) | 1 (0 - 1) | 0.11 (0.05 - 0.18) | -2.60 (-4.45 to -0.72) |
| Tonga | 1 (1 - 2) | 1.04 (0.87 - 1.24) | 0 (0 - 0) | 0.05 (0.02 - 0.08) | -2.18 (-4.05 to -0.26) |
| Vanuatu | 3 (2 - 4) | 1.20 (0.93 - 1.51) | 0 (0 - 0) | 0.04 (0.02 - 0.06) | -3.00 (-5.05 to -0.90) |
| Armenia | 94 (83 - 108) | 2.58 (2.27 - 2.94) | 13 (4 - 26) | 0.62 (0.19 - 1.21) | -2.84 (-3.59 to -2.09) |
| Azerbaijan | 698 (596 - 804) | 7.92 (6.76 - 9.12) | 13 (3 - 31) | 0.20 (0.04 - 0.46) | -4.56 (-6.28 to -2.81) |
| Georgia | 147 (128 - 167) | 3.41 (2.97 - 3.91) | 0 (0 - 1) | 0.01 (0.00 - 0.02) | -9.17 (-11.57 to -6.70) |
| Kazakhstan | 481 (431 - 540) | 2.68 (2.40 - 3.00) | 18 (7 - 36) | 0.10 (0.04 - 0.19) | -6.30 (-7.49 to -5.08) |
| Kyrgyzstan | 311 (278 - 349) | 4.94 (4.41 - 5.53) | 16 (5 - 38) | 0.22 (0.07 - 0.50) | -6.78 (-7.60 to -5.97) |
| Mongolia | 181 (149 - 213) | 5.35 (4.41 - 6.30) | 1 (0 - 4) | 0.03 (0.00 - 0.12) | -7.16 (-9.28 to -4.99) |
| Tajikistan | 543 (478 - 619) | 5.65 (4.99 - 6.43) | 38 (17 - 73) | 0.29 (0.13 - 0.55) | -3.46 (-4.88 to -2.03) |
| Turkmenistan | 331 (292 - 381) | 5.61 (4.96 - 6.45) | 17 (7 - 30) | 0.32 (0.14 - 0.57) | -3.62 (-5.02 to -2.21) |
| Uzbekistan | 1,558 (1,426 - 1,712) | 4.63 (4.24 - 5.08) | 105 (47 - 185) | 0.28 (0.12 - 0.49) | -3.01 (-4.52 to -1.48) |
| Albania | 99 (84 - 115) | 2.63 (2.24 - 3.04) | 0 (0 - 0) | 0.01 (0.00 - 0.02) | -6.73 (-9.24 to -4.15) |
| Bosnia and Herzegovina | 8 (7 - 9) | 0.23 (0.20 - 0.28) | 0 (0 - 0) | 0.00 (0.00 - 0.00) | -3.93 (-6.61 to -1.16) |

| location | Num_1990 | ASDR_1990 | Num_2021 | ASDR_2021 | EAPC_ASDR |
|---|---|---|---|---|---|
| Bulgaria | 77 (73 - 81) | 1.30 (1.23 - 1.39) | 0 (0 - 1) | 0.01 (0.00 - 0.02) | -5.49 (-8.07 to -2.85) |
| Croatia | 13 (12 - 14) | 0.35 (0.33 - 0.38) | 0 (0 - 0) | 0.00 (0.00 - 0.01) | -3.74 (-6.35 to -1.05) |
| Czechia | 38 (36 - 41) | 0.46 (0.43 - 0.49) | 1 (0 - 2) | 0.01 (0.00 - 0.01) | -2.67 (-5.30 - 0.04) |
| Hungary | 23 (21 - 24) | 0.30 (0.28 - 0.33) | 0 (0 - 1) | 0.00 (0.00 - 0.01) | -3.69 (-6.35 to -0.96) |
| North Macedonia | 15 (13 - 19) | 0.93 (0.76 - 1.15) | 0 (0 - 0) | 0.00 (0.00 - 0.00) | -6.92 (-9.56 to -4.20) |
| Montenegro | 2 (1 - 2) | 0.35 (0.28 - 0.45) | 0 (0 - 0) | 0.00 (0.00 - 0.00) | -4.85 (-7.50 to -2.13) |
| Poland | 92 (87 - 96) | 0.29 (0.28 - 0.31) | 5 (1 - 17) | 0.01 (0.00 - 0.03) | -0.64 (-3.01 - 1.80) |
| Romania | 374 (351 - 394) | 2.32 (2.17 - 2.45) | 1 (0 - 4) | 0.01 (0.00 - 0.02) | -5.71 (-8.57 to -2.76) |
| Serbia | 36 (29 - 49) | 0.52 (0.42 - 0.71) | 0 (0 - 1) | 0.00 (0.00 - 0.01) | -5.72 (-8.23 to -3.14) |
| Slovakia | 44 (39 - 48) | 0.98 (0.88 - 1.08) | 1 (0 - 2) | 0.01 (0.00 - 0.03) | -3.25 (-5.69 to -0.74) |
| Slovenia | 8 (7 - 9) | 0.51 (0.47 - 0.55) | 0 (0 - 0) | 0.00 (0.00 - 0.01) | -3.94 (-6.61 to -1.18) |
| Belarus | 41 (36 - 48) | 0.56 (0.48 - 0.65) | 5 (1 - 13) | 0.06 (0.02 - 0.14) | -6.16 (-7.18 to -5.14) |
| Estonia | 5 (5 - 6) | 0.44 (0.40 - 0.48) | 0 (0 - 0) | 0.00 (0.00 - 0.01) | -5.28 (-7.81 to -2.68) |
| Latvia | 8 (7 - 8) | 0.37 (0.34 - 0.40) | 0 (0 - 0) | 0.00 (0.00 - 0.01) | -5.28 (-8.56 to -1.88) |
| Lithuania | 8 (7 - 9) | 0.27 (0.25 - 0.30) | 4 (2 - 7) | 0.12 (0.05 - 0.24) | -1.07 (-1.91 to -0.22) |
| Moldova | 62 (55 - 71) | 1.58 (1.39 - 1.81) | 2 (1 - 4) | 0.09 (0.03 - 0.18) | -3.57 (-5.07 to -2.04) |
| Russia | 760 (728 - 793) | 0.72 (0.69 - 0.75) | 50 (13 - 114) | 0.04 (0.01 - 0.09) | -4.43 (-5.76 to -3.08) |
| Ukraine | 189 (166 - 215) | 0.53 (0.46 - 0.61) | 8 (4 - 13) | 0.02 (0.01 - 0.04) | -3.22 (-4.82 to -1.59) |
| Brunei | 1 (1 - 1) | 0.45 (0.40 - 0.52) | 0 (0 - 0) | 0.00 (0.00 - 0.00) | -2.92 (-7.21 - 1.58) |
| Japan | 646 (580 - 706) | 0.48 (0.43 - 0.52) | 2 (0 - 12) | 0.00 (0.00 - 0.00) | -5.72 (-9.56 to -1.71) |
| South | 93 (79 - 110) | 0.36 (0.30 - | 0 (0 - 0) | 0.00 (0.00 - | -5.23 |

| location | Num_1990 | ASDR_1990 | Num_2021 | ASDR_2021 | EAPC_ASDR |
|---|---|---|---|---|---|
| Korea | | 0.43) | | 0.00) | (-10.18 to -0.01) |
| Singapore | 18 (17 - 19) | 0.93 (0.86 - 1.00) | 0 (0 - 1) | 0.00 (0.00 - 0.01) | -4.44 (-7.79 to -0.97) |
| Australia | 29 (26 - 31) | 0.19 (0.17 - 0.20) | 0 (0 - 1) | 0.00 (0.00 - 0.00) | -4.69 (-8.08 to -1.17) |
| New Zealand | 14 (13 - 16) | 0.45 (0.41 - 0.49) | 0 (0 - 0) | 0.00 (0.00 - 0.00) | -5.75 (-8.98 to -2.40) |
| Andorra | 0 (0 - 0) | 0.21 (0.16 - 0.28) | 0 (0 - 0) | 0.00 (0.00 - 0.01) | -3.31 (-5.45 to -1.12) |
| Austria | 15 (13 - 16) | 0.15 (0.14 - 0.16) | 1 (0 - 2) | 0.00 (0.00 - 0.01) | -3.16 (-5.03 to -1.26) |
| Belgium | 38 (34 - 41) | 0.30 (0.27 - 0.32) | 8 (3 - 16) | 0.03 (0.01 - 0.06) | -1.39 (-2.95 - 0.19) |
| Cyprus | 2 (1 - 2) | 0.36 (0.30 - 0.41) | 0 (0 - 0) | 0.01 (0.00 - 0.01) | -4.13 (-6.13 to -2.09) |
| Denmark | 19 (17 - 21) | 0.27 (0.25 - 0.29) | 1 (0 - 2) | 0.01 (0.00 - 0.01) | -2.66 (-5.19 to -0.06) |
| Finland | 26 (23 - 29) | 0.41 (0.37 - 0.45) | 1 (0 - 1) | 0.01 (0.00 - 0.01) | -8.26 (-10.06 to -6.43) |
| France | 189 (168 - 207) | 0.25 (0.22 - 0.27) | 19 (7 - 39) | 0.01 (0.00 - 0.02) | -3.18 (-4.94 to -1.37) |
| Germany | 199 (180 - 216) | 0.20 (0.18 - 0.21) | 4 (1 - 9) | 0.00 (0.00 - 0.00) | -2.83 (-5.64 - 0.07) |
| Greece | 23 (21 - 24) | 0.20 (0.19 - 0.22) | 0 (0 - 1) | 0.00 (0.00 - 0.00) | -1.37 (-5.17 - 2.58) |
| Iceland | 1 (1 - 1) | 0.43 (0.38 - 0.47) | 1 (1 - 2) | 0.17 (0.08 - 0.25) | -2.82 (-3.61 to -2.03) |
| Ireland | 20 (19 - 22) | 0.59 (0.53 - 0.63) | 1 (0 - 4) | 0.02 (0.00 - 0.05) | -4.37 (-6.21 to -2.49) |
| Israel | 9 (9 - 10) | 0.21 (0.20 - 0.23) | 1 (0 - 2) | 0.01 (0.00 - 0.01) | -2.10 (-4.22 - 0.06) |
| Italy | 101 (91 - 110) | 0.19 (0.17 - 0.20) | 9 (4 - 15) | 0.01 (0.00 - 0.01) | -2.80 (-4.72 to -0.85) |
| Luxembourg | 1 (1 - 1) | 0.22 (0.20 - 0.23) | 0 (0 - 0) | 0.01 (0.00 - 0.01) | -3.34 (-5.47 to -1.17) |
| Malta | 1 (1 - 1) | 0.30 (0.28 - 0.33) | 0 (0 - 0) | 0.01 (0.01 - 0.02) | -2.29 (-4.30 to -0.25) |
| Netherlands | 46 (41 - 51) | 0.27 (0.24 - 0.29) | 4 (1 - 12) | 0.01 (0.00 - 0.04) | -3.13 (-5.26 to -0.96) |
| Norway | 30 (26 - 33) | 0.43 (0.38 - | 1 (0 - 3) | 0.01 (0.00 - | -4.67 (-6.76 |

| location | Num_1990 | ASDR_1990 | Num_2021 | ASDR_2021 | EAPC_ASDR |
|---|---|---|---|---|---|
| | | 0.47) | | 0.02) | to -2.53) |
| Portugal | 36 (33 - 38) | 0.40 (0.37 - 0.42) | 6 (1 - 19) | 0.02 (0.00 - 0.07) | -1.56 (-3.50 - 0.42) |
| Spain | 98 (88 - 107) | 0.24 (0.22 - 0.26) | 12 (3 - 33) | 0.01 (0.00 - 0.03) | -2.12 (-3.99 to -0.21) |
| Sweden | 46 (40 - 51) | 0.31 (0.28 - 0.34) | 7 (2 - 16) | 0.03 (0.01 - 0.06) | -4.15 (-5.22 to -3.06) |
| Switzerland | 31 (27 - 34) | 0.33 (0.30 - 0.36) | 1 (1 - 2) | 0.01 (0.00 - 0.01) | -4.75 (-6.61 to -2.85) |
| UK | 359 (322 - 392) | 0.45 (0.41 - 0.48) | 21 (11 - 36) | 0.02 (0.01 - 0.03) | -3.86 (-5.91 to -1.77) |
| Argentina | 155 (146 - 165) | 0.49 (0.46 - 0.52) | 29 (11 - 58) | 0.06 (0.02 - 0.12) | -0.03 (-2.24 - 2.23) |
| Chile | 108 (102 - 114) | 0.92 (0.86 - 0.97) | 2 (1 - 5) | 0.01 (0.00 - 0.02) | -6.43 (-8.87 to -3.93) |
| Uruguay | 14 (13 - 15) | 0.47 (0.44 - 0.50) | 1 (0 - 2) | 0.02 (0.01 - 0.04) | -3.12 (-5.48 to -0.70) |
| Canada | 92 (82 - 99) | 0.33 (0.29 - 0.35) | 7 (2 - 18) | 0.01 (0.00 - 0.03) | -3.80 (-5.58 to -1.98) |
| USA | 876 (787 - 944) | 0.30 (0.27 - 0.32) | 107 (48 - 193) | 0.02 (0.01 - 0.04) | -3.20 (-4.54 to -1.85) |
| Antigua and Barbuda | 0 (0 - 0) | 0.44 (0.38 - 0.49) | 0 (0 - 0) | 0.00 (0.00 - 0.02) | -1.76 (-5.20 - 1.80) |
| Bahamas | 1 (1 - 2) | 0.64 (0.55 - 0.74) | 0 (0 - 0) | 0.00 (0.00 - 0.01) | -2.94 (-6.36 - 0.60) |
| Barbados | 1 (1 - 1) | 0.43 (0.38 - 0.47) | 0 (0 - 0) | 0.00 (0.00 - 0.01) | -2.20 (-5.70 - 1.43) |
| Belize | 3 (2 - 3) | 1.02 (0.92 - 1.14) | 0 (0 - 0) | 0.00 (0.00 - 0.02) | -4.21 (-7.54 to -0.76) |
| Cuba | 47 (44 - 50) | 0.53 (0.50 - 0.56) | 0 (0 - 2) | 0.00 (0.00 - 0.02) | -2.34 (-5.66 - 1.10) |
| Dominica | 0 (0 - 0) | 0.49 (0.42 - 0.57) | 0 (0 - 0) | 0.00 (0.00 - 0.02) | -0.95 (-4.34 - 2.57) |
| Dominican Republic | 88 (72 - 105) | 0.92 (0.76 - 1.08) | 0 (0 - 1) | 0.00 (0.00 - 0.01) | -4.97 (-8.29 to -1.53) |
| Grenada | 1 (1 - 1) | 0.78 (0.67 - 0.90) | 0 (0 - 0) | 0.00 (0.00 - 0.02) | -2.75 (-6.22 - 0.86) |
| Guyana | 8 (7 - 10) | 0.83 (0.71 - | 0 (0 - 0) | 0.00 (0.00 - | -2.40 (-5.84 |

| location | Num_1990 | ASDR_1990 | Num_2021 | ASDR_2021 | EAPC_ASDR |
|---|---|---|---|---|---|
| | | 0.95) | | 0.02) | - 1.17) |
| Haiti | 407 (324 - 510) | 3.69 (2.97 - 4.56) | 1 (0 - 7) | 0.01 (0.00 - 0.05) | -4.88 (-8.05 to -1.60) |
| Jamaica | 9 (8 - 10) | 0.37 (0.34 - 0.41) | 0 (0 - 0) | 0.00 (0.00 - 0.01) | -3.61 (-6.89 to -0.21) |
| Saint Lucia | 1 (1 - 1) | 0.51 (0.45 - 0.58) | 0 (0 - 0) | 0.00 (0.00 - 0.01) | -3.36 (-6.64 - 0.04) |
| Saint Vincent and the Grenadines | 1 (0 - 1) | 0.56 (0.48 - 0.65) | 0 (0 - 0) | 0.00 (0.00 - 0.01) | -3.18 (-6.49 - 0.25) |
| Suriname | 3 (3 - 4) | 0.82 (0.67 - 0.96) | 0 (0 - 0) | 0.00 (0.00 - 0.02) | -3.70 (-6.97 to -0.32) |
| Trinidad and Tobago | 6 (5 - 6) | 0.57 (0.51 - 0.63) | 0 (0 - 0) | 0.00 (0.00 - 0.01) | -4.10 (-7.43 to -0.66) |
| Bolivia (Plurinational State of) | 384 (278 - 490) | 3.88 (2.86 - 4.89) | 4 (1 - 10) | 0.04 (0.01 - 0.09) | -5.09 (-7.63 to -2.48) |
| Ecuador | 161 (144 - 178) | 1.30 (1.18 - 1.42) | 21 (4 - 57) | 0.14 (0.03 - 0.37) | -1.86 (-3.34 to -0.36) |
| Peru | 859 (740 - 1,006) | 3.06 (2.66 - 3.55) | 259 (83 - 439) | 0.78 (0.25 - 1.32) | -3.37 (-4.62 to -2.10) |
| Colombia | 367 (319 - 418) | 0.92 (0.81 - 1.03) | 19 (4 - 49) | 0.05 (0.01 - 0.12) | -2.98 (-4.70 to -1.23) |
| Costa Rica | 17 (16 - 19) | 0.51 (0.47 - 0.55) | 0 (0 - 1) | 0.01 (0.00 - 0.02) | -3.69 (-6.25 to -1.05) |
| El Salvador | 82 (68 - 96) | 1.10 (0.93 - 1.28) | 1 (0 - 2) | 0.01 (0.00 - 0.03) | -4.65 (-7.48 to -1.73) |
| Guatemala | 311 (280 - 346) | 2.48 (2.29 - 2.72) | 55 (21 - 111) | 0.39 (0.15 - 0.80) | -0.71 (-2.09 - 0.68) |
| Honduras | 106 (87 - 125) | 1.33 (1.11 - 1.57) | 21 (6 - 48) | 0.22 (0.06 - 0.51) | -2.50 (-3.49 to -1.49) |
| Mexico | 1,623 (1,475 - 1,818) | 1.47 (1.35 - 1.63) | 86 (43 - 147) | 0.09 (0.04 - 0.15) | -3.51 (-4.80 to -2.21) |
| Nicaragua | 99 (85 - 116) | 1.56 (1.34 - 1.82) | 5 (2 - 11) | 0.09 (0.03 - 0.17) | -3.56 (-5.88 to -1.19) |
| Panama | 16 (13 - 18) | 0.61 (0.53 - | 9 (2 - 21) | 0.24 (0.05 - | 0.02 (-1.37 |

| location | Num_1990 | ASDR_1990 | Num_2021 | ASDR_2021 | EAPC_ASDR |
|---|---|---|---|---|---|
| | | 0.69) | | 0.57) | - 1.43) |
| Venezuela | 159 (149 - 171) | 0.71 (0.66 - 0.75) | 17 (9 - 29) | 0.07 (0.04 - 0.12) | -0.71 (-2.42 - 1.03) |
| Brazil | 2,020 (1,804 - 2,245) | 1.39 (1.25 - 1.54) | 260 (76 - 584) | 0.13 (0.04 - 0.28) | -3.25 (-4.55 to -1.93) |
| Paraguay | 45 (36 - 57) | 0.80 (0.65 - 0.99) | 3 (1 - 8) | 0.05 (0.01 - 0.12) | -2.69 (-4.64 to -0.70) |
| Algeria | 351 (259 - 482) | 1.06 (0.81 - 1.41) | 43 (20 - 79) | 0.11 (0.05 - 0.21) | -2.82 (-3.83 to -1.80) |
| Bahrain | 3 (2 - 3) | 0.59 (0.51 - 0.69) | 1 (0 - 1) | 0.08 (0.03 - 0.15) | -1.32 (-2.62 - 0.00) |
| Egypt | 3,642 (3,008 - 4,496) | 4.24 (3.52 - 5.20) | 304 (99 - 648) | 0.29 (0.10 - 0.62) | -4.75 (-5.57 to -3.92) |
| Iran | 839 (693 - 1,107) | 1.16 (0.97 - 1.50) | 92 (48 - 133) | 0.14 (0.07 - 0.21) | -4.75 (-5.30 to -4.20) |
| Iraq | 376 (297 - 478) | 1.20 (0.95 - 1.51) | 47 (20 - 88) | 0.12 (0.05 - 0.23) | -3.73 (-4.63 to -2.82) |
| Jordan | 49 (41 - 59) | 0.89 (0.74 - 1.04) | 10 (3 - 24) | 0.11 (0.03 - 0.26) | -2.23 (-3.30 to -1.15) |
| Kuwait | 9 (8 - 10) | 0.59 (0.53 - 0.65) | 5 (2 - 9) | 0.20 (0.09 - 0.38) | 1.80 (0.51 - 3.10) |
| Lebanon | 42 (30 - 57) | 1.11 (0.82 - 1.47) | 9 (4 - 17) | 0.18 (0.09 - 0.33) | -1.91 (-2.90 to -0.91) |
| Libya | 35 (27 - 47) | 0.64 (0.50 - 0.82) | 5 (2 - 11) | 0.12 (0.05 - 0.25) | -0.05 (-1.29 - 1.19) |
| Morocco | 816 (624 - 1,039) | 2.25 (1.73 - 2.86) | 46 (19 - 88) | 0.15 (0.06 - 0.28) | -4.57 (-5.56 to -3.58) |
| Palestine | 35 (27 - 45) | 0.99 (0.79 - 1.22) | 6 (3 - 11) | 0.12 (0.06 - 0.23) | -2.37 (-3.40 to -1.34) |
| Oman | 28 (20 - 42) | 0.98 (0.75 - 1.40) | 4 (2 - 8) | 0.14 (0.05 - 0.28) | -1.57 (-3.08 to -0.03) |
| Qatar | 2 (1 - 2) | 0.44 (0.36 - 0.54) | 0 (0 - 0) | 0.01 (0.00 - 0.03) | -2.95 (-5.27 to -0.58) |
| Saudi Arabia | 190 (146 - 255) | 0.95 (0.76 - 1.22) | 20 (8 - 37) | 0.10 (0.04 - 0.18) | -2.87 (-3.95 to -1.78) |
| Syria | 183 (138 - 255) | 0.91 (0.70 - 1.23) | 11 (5 - 21) | 0.11 (0.04 - 0.21) | -0.59 (-2.16 - 1.00) |
| Tunisia | 151 (103 - 239) | 1.50 (1.04 - 2.34) | 10 (4 - 18) | 0.10 (0.04 - 0.19) | -4.69 (-5.61 to -3.76) |
| Turkey | 1,370 (1,042 - 1,840) | 2.01 (1.54 - 2.68) | 66 (30 - 119) | 0.10 (0.05 - 0.19) | -5.66 (-6.58 to -4.74) |
| United | 10 (7 - 13) | 0.62 (0.50 - | 2 (1 - 4) | 0.09 (0.04 - | -0.41 (-1.90 |

| location | Num_1990 | ASDR_1990 | Num_2021 | ASDR_2021 | EAPC_ASDR |
|---|---|---|---|---|---|
| Arab Emirates | | 0.78) | | 0.16) | - 1.10) |
| Yemen | 721 (546 - 1,017) | 2.53 (1.94 - 3.51) | 97 (43 - 180) | 0.23 (0.11 - 0.43) | -3.64 (-4.58 to -2.68) |
| Afghanistan | 785 (600 - 1,045) | 4.12 (3.19 - 5.44) | 162 (71 - 321) | 0.32 (0.14 - 0.62) | -4.04 (-5.06 to -3.02) |
| Bangladesh | 6,581 (5,522 - 7,768) | 3.43 (2.90 - 4.03) | 743 (325 - 1,378) | 0.55 (0.24 - 1.02) | -4.03 (-4.86 to -3.19) |
| Bhutan | 25 (17 - 34) | 2.55 (1.72 - 3.42) | 3 (1 - 5) | 0.47 (0.21 - 0.87) | -4.00 (-4.64 to -3.36) |
| India | 28,106 (22,399 - 33,705) | 2.47 (1.99 - 2.95) | 8,294 (3,079 - 15,045) | 0.79 (0.29 - 1.42) | -2.21 (-2.88 to -1.53) |
| Nepal | 1,663 (1,352 - 2,009) | 4.74 (3.86 - 5.71) | 153 (57 - 309) | 0.52 (0.19 - 1.04) | -4.66 (-5.33 to -3.99) |
| Pakistan | 4,728 (3,744 - 5,841) | 2.46 (1.97 - 3.02) | 2,368 (1,308 - 3,811) | 0.83 (0.46 - 1.33) | -0.63 (-1.60 - 0.35) |
| Angola | 737 (558 - 952) | 3.66 (2.84 - 4.66) | 369 (166 - 609) | 0.88 (0.42 - 1.39) | -3.88 (-4.16 to -3.60) |
| Central African Republic | 241 (164 - 325) | 4.61 (3.28 - 6.10) | 136 (63 - 228) | 1.78 (0.86 - 2.90) | -2.12 (-2.38 to -1.86) |
| Congo (Brazzaville) | 66 (46 - 90) | 1.86 (1.36 - 2.45) | 30 (14 - 48) | 0.67 (0.32 - 1.04) | -2.56 (-2.84 to -2.28) |
| DR Congo | 2,363 (1,609 - 3,158) | 3.24 (2.30 - 4.21) | 983 (467 - 1,606) | 0.98 (0.48 - 1.54) | -2.90 (-3.23 to -2.58) |
| Equatorial Guinea | 26 (18 - 34) | 3.08 (2.22 - 4.03) | 8 (3 - 14) | 0.67 (0.31 - 1.12) | -4.87 (-5.17 to -4.57) |
| Gabon | 27 (19 - 37) | 1.89 (1.38 - 2.47) | 11 (5 - 19) | 0.71 (0.32 - 1.19) | -2.11 (-2.38 to -1.84) |
| Burundi | 307 (223 - 400) | 2.94 (2.17 - 3.70) | 137 (77 - 229) | 0.79 (0.48 - 1.28) | -2.30 (-3.14 to -1.45) |
| Comoros | 29 (23 - 38) | 3.48 (2.68 - 4.48) | 7 (4 - 10) | 0.91 (0.58 - 1.30) | -3.02 (-3.60 to -2.43) |
| Djibouti | 13 (9 - 17) | 2.06 (1.55 - 2.64) | 7 (4 - 11) | 0.65 (0.40 - 0.99) | -2.17 (-2.93 to -1.40) |
| Eritrea | 162 (123 - 207) | 2.79 (2.12 - 3.51) | 83 (51 - 127) | 1.13 (0.71 - 1.77) | -1.23 (-1.91 to -0.56) |
| Ethiopi | 3,526 (2,657 - | 3.69 (2.86 - | 884 (573 - | 0.67 (0.43 - | -4.38 (-4.93 |

| location | Num_1990 | ASDR_1990 | Num_2021 | ASDR_2021 | EAPC_ASDR |
|---|---|---|---|---|---|
| a | 4,441) | 4.55) | 1,329) | 1.01) | to -3.82) |
| Kenya | 987 (815 - 1,267) | 2.41 (2.03 - 2.99) | 355 (218 - 532) | 0.78 (0.48 - 1.18) | -1.38 (-2.29 to -0.45) |
| Madagascar | 810 (697 - 940) | 3.65 (3.18 - 4.20) | 293 (102 - 579) | 0.82 (0.29 - 1.64) | -2.32 (-3.19 to -1.45) |
| Malawi | 723 (526 - 960) | 3.69 (2.75 - 4.76) | 238 (137 - 365) | 1.04 (0.62 - 1.57) | -2.36 (-3.12 to -1.58) |
| Mauritius | 7 (6 - 7) | 0.74 (0.70 - 0.79) | 0 (0 - 1) | 0.03 (0.02 - 0.05) | -1.56 (-3.54 - 0.45) |
| Mozambique | 651 (480 - 956) | 2.64 (1.98 - 3.79) | 310 (191 - 480) | 0.76 (0.47 - 1.14) | -2.11 (-2.82 to -1.40) |
| Rwanda | 494 (365 - 638) | 3.78 (2.84 - 4.82) | 122 (73 - 186) | 0.82 (0.51 - 1.25) | -3.73 (-4.47 to -2.98) |
| Seychelles | 1 (1 - 1) | 1.00 (0.90 - 1.12) | 0 (0 - 0) | 0.05 (0.03 - 0.09) | -0.64 (-2.72 - 1.49) |
| Somalia | 557 (387 - 752) | 3.57 (2.56 - 4.74) | 337 (205 - 543) | 0.93 (0.58 - 1.47) | -2.70 (-3.40 to -2.00) |
| Tanzania | 2,122 (1,669 - 2,599) | 4.28 (3.39 - 5.19) | 906 (509 - 1,479) | 1.16 (0.67 - 1.87) | -2.98 (-3.63 to -2.31) |
| Uganda | 633 (444 - 906) | 1.82 (1.34 - 2.56) | 347 (128 - 742) | 0.62 (0.23 - 1.26) | -1.58 (-2.41 to -0.75) |
| Zambia | 543 (428 - 696) | 3.51 (2.80 - 4.43) | 274 (153 - 418) | 1.16 (0.67 - 1.75) | -2.20 (-2.70 to -1.70) |
| Botswana | 27 (21 - 33) | 1.61 (1.29 - 1.97) | 6 (2 - 12) | 0.30 (0.11 - 0.58) | -1.26 (-2.99 - 0.50) |
| Lesotho | 42 (35 - 52) | 1.90 (1.59 - 2.30) | 10 (4 - 20) | 0.56 (0.21 - 1.05) | 0.21 (-1.42 - 1.87) |
| Namibia | 28 (22 - 35) | 1.46 (1.17 - 1.81) | 6 (2 - 12) | 0.26 (0.10 - 0.52) | -1.18 (-2.87 - 0.55) |
| South Africa | 1,026 (882 - 1,226) | 2.24 (1.95 - 2.66) | 126 (51 - 253) | 0.27 (0.11 - 0.54) | -2.15 (-4.12 to -0.14) |
| Eswatini | 23 (19 - 29) | 1.87 (1.53 - 2.25) | 4 (2 - 8) | 0.35 (0.14 - 0.69) | -0.96 (-2.76 - 0.88) |
| Zimbabwe | 333 (284 - 390) | 2.19 (1.91 - 2.54) | 92 (36 - 181) | 0.48 (0.19 - 0.95) | -0.48 (-2.19 - 1.26) |
| Benin | 357 (281 - 441) | 3.68 (2.95 - 4.51) | 237 (136 - 365) | 1.13 (0.65 - 1.71) | -1.49 (-2.15 to -0.82) |
| Burkina Faso | 969 (733 - 1,210) | 5.02 (3.83 - 6.20) | 700 (405 - 1,060) | 1.79 (1.04 - 2.68) | -1.00 (-1.66 to -0.35) |
| Cameroon | 569 (438 - 743) | 2.93 (2.30 - 3.77) | 450 (205 - 778) | 1.07 (0.50 - 1.81) | -1.05 (-1.79 to -0.31) |
| Cape | 6 (4 - 8) | 1.10 (0.86 - | 2 (1 - 3) | 0.42 (0.26 - | -2.00 (-2.56 |

| location | Num_1990 | ASDR_1990 | Num_2021 | ASDR_2021 | EAPC_ASDR |
|---|---|---|---|---|---|
| Verde | | 1.41) | | 0.67) | to -1.43) |
| Chad | 515 (407 - 635) | 4.01 (3.23 - 4.88) | 529 (332 - 796) | 1.54 (0.98 - 2.29) | -1.30 (-1.85 to -0.74) |
| Côte d'Ivoire | 808 (622 - 1,072) | 3.53 (2.78 - 4.60) | 280 (143 - 464) | 0.72 (0.36 - 1.17) | -1.59 (-2.48 to -0.69) |
| The Gambia | 53 (42 - 67) | 3.02 (2.47 - 3.76) | 22 (14 - 32) | 0.81 (0.52 - 1.21) | -2.60 (-3.19 to -2.01) |
| Ghana | 491 (381 - 627) | 2.06 (1.64 - 2.59) | 256 (139 - 416) | 0.71 (0.41 - 1.13) | -0.95 (-1.67 to -0.22) |
| Guinea | 701 (535 - 891) | 5.79 (4.43 - 7.39) | 241 (137 - 367) | 1.21 (0.70 - 1.82) | -2.86 (-3.48 to -2.24) |
| Guinea-Bissau | 62 (47 - 79) | 3.48 (2.71 - 4.35) | 17 (11 - 26) | 0.73 (0.46 - 1.08) | -3.04 (-3.63 to -2.43) |
| Liberia | 218 (164 - 287) | 4.61 (3.46 - 6.04) | 41 (24 - 65) | 0.66 (0.40 - 1.03) | -4.45 (-5.23 to -3.66) |
| Mali | 418 (329 - 532) | 2.39 (1.89 - 3.02) | 381 (228 - 569) | 0.90 (0.54 - 1.34) | -1.14 (-1.94 to -0.33) |
| Mauritania | 84 (67 - 102) | 2.38 (1.95 - 2.85) | 36 (23 - 55) | 0.69 (0.44 - 1.03) | -2.14 (-2.72 to -1.56) |
| Niger | 1,150 (864 - 1,505) | 6.63 (4.99 - 8.60) | 618 (359 - 944) | 1.31 (0.77 - 1.98) | -3.57 (-4.17 to -2.97) |
| Nigeria | 9,113 (7,429 - 10,811) | 5.40 (4.40 - 6.41) | 5,774 (3,300 - 8,860) | 1.61 (0.93 - 2.46) | -1.86 (-2.46 to -1.27) |
| São Tomé and Príncipe | 6 (4 - 7) | 2.94 (2.40 - 3.46) | 1 (1 - 2) | 0.58 (0.35 - 0.93) | -3.44 (-4.03 to -2.85) |
| Senegal | 397 (321 - 477) | 2.75 (2.25 - 3.30) | 157 (79 - 279) | 0.79 (0.41 - 1.38) | -2.13 (-2.82 to -1.43) |
| Sierra Leone | 407 (300 - 540) | 4.98 (3.69 - 6.52) | 117 (67 - 195) | 1.00 (0.58 - 1.64) | -3.45 (-4.00 to -2.89) |
| Togo | 194 (153 - 244) | 3.01 (2.41 - 3.69) | 136 (74 - 213) | 1.43 (0.80 - 2.18) | -1.24 (-1.67 to -0.80) |
| American Samoa | 0 (0 - 1) | 0.75 (0.65 - 0.86) | 0 (0 - 0) | 0.03 (0.01 - 0.04) | -3.08 (-4.95 to -1.17) |
| Bermuda | 0 (0 - 0) | 0.28 (0.25 - 0.31) | 0 (0 - 0) | 0.00 (0.00 - 0.00) | -4.77 (-7.96 to -1.47) |
| Cook Islands | 0 (0 - 0) | 1.78 (1.48 - 2.11) | 0 (0 - 0) | 0.04 (0.02 - 0.07) | -5.70 (-7.20 to -4.19) |
| Greenla | 0 (0 - 0) | 0.61 (0.50 - | 0 (0 - 0) | 0.03 (0.01 - | -3.74 (-5.28 |

| location | Num_1990 | ASDR_1990 | Num_2021 | ASDR_2021 | EAPC_ASDR |
|---|---|---|---|---|---|
| nd | | 0.73) | | 0.04) | to -2.17) |
| Guam | 1 (1 - 1) | 0.57 (0.50 - 0.64) | 0 (0 - 0) | 0.02 (0.01 - 0.03) | -2.49 (-4.63 to -0.31) |
| Monaco | 0 (0 - 0) | 0.29 (0.22 - 0.35) | 0 (0 - 0) | 0.02 (0.01 - 0.03) | -1.85 (-3.84 - 0.19) |
| Nauru | 0 (0 - 0) | 1.81 (1.46 - 2.18) | 0 (0 - 0) | 0.11 (0.05 - 0.21) | -1.39 (-3.45 - 0.72) |
| Niue | 0 (0 - 0) | 1.26 (1.02 - 1.54) | 0 (0 - 0) | 0.25 (0.13 - 0.42) | 0.42 (-1.11 - 1.96) |
| Northern Mariana Islands | 0 (0 - 0) | 0.58 (0.47 - 0.71) | 0 (0 - 0) | 0.02 (0.01 - 0.03) | -3.16 (-5.19 to -1.08) |
| Palau | 0 (0 - 0) | 1.54 (1.17 - 1.96) | 0 (0 - 0) | 0.07 (0.03 - 0.12) | -1.72 (-3.71 - 0.31) |
| Puerto Rico | 15 (14 - 16) | 0.48 (0.45 - 0.51) | 0 (0 - 0) | 0.00 (0.00 - 0.01) | -5.10 (-8.47 to -1.61) |
| Saint Kitts and Nevis | 0 (0 - 0) | 0.81 (0.74 - 0.88) | 0 (0 - 0) | 0.00 (0.00 - 0.02) | -2.77 (-6.11 - 0.69) |
| San Marino | 0 (0 - 0) | 0.20 (0.16 - 0.24) | 0 (0 - 0) | 0.00 (0.00 - 0.01) | -3.75 (-5.96 to -1.48) |
| Tokelau | 0 (0 - 0) | 1.19 (0.91 - 1.48) | 0 (0 - 0) | 0.13 (0.06 - 0.23) | -2.99 (-4.08 to -1.89) |
| Tuvalu | 1 (0 - 1) | 3.32 (2.61 - 4.23) | 0 (0 - 0) | 0.05 (0.03 - 0.09) | -5.39 (-7.18 to -3.57) |
| Virgin Islands | 0 (0 - 0) | 0.31 (0.26 - 0.37) | 0 (0 - 0) | 0.00 (0.00 - 0.00) | -4.78 (-8.18 to -1.26) |
| South Sudan | 394 (304 - 520) | 3.76 (2.93 - 4.89) | 201 (117 - 357) | 1.34 (0.79 - 2.26) | -1.80 (-2.50 to -1.09) |
| Sudan | 1,130 (830 - 1,544) | 3.07 (2.28 - 4.19) | 100 (44 - 188) | 0.20 (0.09 - 0.37) | -4.71 (-5.69 to -3.72) |

**Table S2.** The death cases and **DALY** of RSV-related LRI in 1990 and 2021, with Temporal Trends from 1990 to 2021 in 204 countries or territories.

| location | Num_1990 | DALY_1990 | Num_2021 | DALY_2021 | EAPC_DALY |
|---|---|---|---|---|---|
| China | 2,231,999 (1,910,836 - 2,586,118) | 202.64 (173.57 - 234.74) | 43,753 (24,591 - 72,959) | 6.70 (3.72 - 11.12) | -8.27 (-9.10 to -7.43) |
| North Korea | 31,303 (23,554 - 40,876) | 125.19 (94.36 - 162.87) | 1,086 (530 - 1,920) | 6.84 (3.29 - 12.22) | -4.86 (-6.06 to -3.64) |
| Taiwan (Province of China) | 5,031 (4,724 - 5,329) | 31.92 (30.02 - 33.83) | 742 (412 - 1,230) | 4.77 (2.62 - 7.94) | -1.83 (-3.18 to -0.46) |
| Cambodia | 104,595 (87,344 - 128,606) | 538.77 (451.41 - 660.11) | 313 (102 - 694) | 1.85 (0.60 - 4.09) | -5.97 (-8.76 to -3.09) |
| Indonesia | 305,999 (254,377 - 376,535) | 138.42 (115.08 - 170.07) | 3,950 (1,516 - 8,408) | 1.84 (0.71 - 3.93) | -5.29 (-7.21 to -3.33) |
| Laos | 35,841 (27,191 - 45,312) | 466.58 (354.87 - 589.32) | 1,749 (742 - 3,359) | 21.20 (8.98 - 40.62) | -5.04 (-6.30 to -3.77) |
| Malaysia | 9,010 (7,263 - 10,826) | 41.24 (34.14 - 48.76) | 2,452 (1,019 - 4,730) | 9.43 (3.93 - 18.16) | -0.26 (-1.34 - 0.84) |
| Maldives | 421 (337 - 539) | 102.82 (82.83 - 131.13) | 4 (2 - 6) | 1.18 (0.59 - 2.05) | -5.68 (-7.61 to -3.70) |
| Myanmar | 225,304 (173,606 - 282,978) | 436.13 (335.94 - 548.50) | 3,712 (1,795 - 6,503) | 7.21 (3.49 - 12.63) | -5.37 (-7.29 to -3.41) |
| Philippines | 164,784 (138,550 - 198,438) | 177.88 (149.95 - 213.62) | 13,070 (6,003 - 25,731) | 12.20 (5.60 - 23.94) | -3.39 (-4.75 to -2.02) |
| Sri Lanka | 5,364 (4,629 - 6,187) | 32.03 (27.76 - 36.85) | 56 (12 - 165) | 0.32 (0.07 - 0.93) | -4.62 (-7.23 to -1.93) |
| Thailand | 32,152 (25,155 - 41,411) | 64.37 (50.45 - 82.76) | 112 (18 - 320) | 0.25 (0.04 - 0.74) | -5.85 (-8.61 to -3.01) |
| Timor-Leste | 5,983 (4,897 - 7,420) | 395.02 (324.39 - 488.87) | 106 (52 - 182) | 5.65 (2.76 - 9.72) | -5.73 (-7.53 to -3.90) |
| Vietnam | 139,257 (114,475 - 174,417) | 151.16 (124.33 - 188.80) | 1,649 (445 - 3,808) | 2.13 (0.57 - 4.91) | -4.97 (-6.92 to -2.98) |
| Fiji | 510 (399 - 645) | 58.01 (45.77 - 72.77) | 27 (13 - 47) | 3.05 (1.49 - 5.37) | -0.99 (-3.27 - 1.35) |
| Kiribati | 120 (90 - 154) | 98.43 (74.71 - 125.30) | 4 (2 - 8) | 2.96 (1.38 - 5.47) | -3.45 (-5.30 to -1.57) |
| Marshall Islands | 63 (50 - 78) | 92.34 (73.24 - 112.69) | 2 (1 - 4) | 4.46 (2.08 - 7.59) | -1.95 (-4.10 - 0.26) |

| location | Num_1990 | DALY_1990 | Num_2021 | DALY_2021 | EAPC_DALY |
|---|---|---|---|---|---|
| Federated States of Micronesia | 190 (148 - 234) | 128.74 (100.97 - 158.16) | 3 (1 - 5) | 3.30 (1.60 - 5.67) | -3.95 (-5.90 to -1.96) |
| Papua New Guinea | 22,380 (17,815 - 28,312) | 326.95 (260.35 - 412.69) | 1,876 (893 - 3,463) | 11.75 (5.60 - 21.65) | -2.34 (-4.37 to -0.27) |
| Samoa | 246 (179 - 321) | 98.52 (72.63 - 127.41) | 7 (3 - 12) | 2.64 (1.25 - 4.44) | -3.76 (-5.58 to -1.91) |
| Solomon Islands | 1,225 (954 - 1,572) | 201.71 (156.86 - 257.14) | 70 (35 - 119) | 7.66 (3.80 - 12.96) | -2.78 (-4.62 to -0.90) |
| Tonga | 114 (90 - 140) | 76.29 (61.26 - 93.49) | 5 (2 - 8) | 3.29 (1.61 - 5.55) | -2.51 (-4.37 to -0.61) |
| Vanuatu | 256 (189 - 328) | 93.16 (69.54 - 118.70) | 11 (5 - 20) | 2.73 (1.29 - 4.81) | -3.16 (-5.24 to -1.04) |
| Armenia | 8,373 (7,361 - 9,577) | 228.13 (200.59 - 260.87) | 888 (277 - 1,735) | 48.83 (15.29 - 95.47) | -3.18 (-3.93 to -2.43) |
| Azerbaijan | 62,345 (53,167 - 71,831) | 705.28 (601.61 - 812.43) | 1,136 (242 - 2,694) | 17.15 (3.65 - 40.68) | -4.61 (-6.33 to -2.86) |
| Georgia | 12,883 (11,202 - 14,752) | 301.56 (261.80 - 345.73) | 13 (3 - 42) | 0.48 (0.11 - 1.56) | -10.04 (-12.43 to -7.59) |
| Kazakhstan | 42,628 (38,132 - 47,824) | 236.28 (211.36 - 265.06) | 1,376 (528 - 2,658) | 6.99 (2.68 - 13.49) | -6.98 (-8.19 to -5.76) |
| Kyrgyzstan | 27,751 (24,723 - 31,080) | 438.16 (390.57 - 490.65) | 1,425 (426 - 3,258) | 18.65 (5.57 - 42.64) | -6.94 (-7.75 to -6.12) |
| Mongolia | 16,059 (13,211 - 18,969) | 470.92 (387.87 - 555.59) | 89 (6 - 385) | 2.40 (0.16 - 10.32) | -7.29 (-9.41 to -5.12) |
| Tajikistan | 48,221 (42,464 - 54,959) | 496.01 (436.88 - 564.84) | 3,353 (1,462 - 6,416) | 24.90 (10.85 - 47.60) | -3.50 (-4.91 to -2.06) |
| Turkmenistan | 29,461 (26,000 - 33,950) | 496.03 (437.95 - 571.39) | 1,476 (661 - 2,653) | 27.66 (12.38 - 49.73) | -3.66 (-5.05 to -2.24) |
| Uzbekistan | 138,852 (127,050 - 152,556) | 410.44 (375.66 - 450.77) | 9,308 (4,149 - 16,340) | 24.26 (10.82 - 42.60) | -3.05 (-4.55 to -1.52) |
| Albania | 8,604 (7,268 - 10,025) | 221.11 (187.08 - 257.45) | 12 (4 - 27) | 0.79 (0.29 - 1.82) | -7.00 (-9.51 to -4.42) |
| Bosnia and Herzeg | 555 (445 - 678) | 16.32 (13.01 - 20.02) | 2 (1 - 5) | 0.11 (0.04 - 0.25) | -4.63 (-7.32 to -1.85) |

| location | Num_1990 | DALY_1990 | Num_2021 | DALY_2021 | EAPC_DALY |
|---|---|---|---|---|---|
| ovina | | | | | |
| Bulgaria | 5,236 (4,933 - 5,618) | 97.58 (91.37 - 105.11) | 19 (7 - 41) | 0.53 (0.20 - 1.13) | -5.64 (-8.22 to -2.98) |
| Croatia | 727 (658 - 796) | 23.47 (20.98 - 26.01) | 6 (2 - 13) | 0.21 (0.08 - 0.44) | -3.71 (-6.36 to -0.99) |
| Czechia | 2,227 (2,066 - 2,396) | 32.16 (29.56 - 34.92) | 24 (9 - 50) | 0.26 (0.10 - 0.54) | -3.87 (-6.48 to -1.20) |
| Hungary | 1,525 (1,398 - 1,668) | 23.18 (21.09 - 25.58) | 9 (4 - 20) | 0.14 (0.05 - 0.29) | -4.33 (-6.99 to -1.59) |
| North Macedonia | 1,315 (1,070 - 1,641) | 79.80 (64.93 - 99.64) | 1 (1 - 3) | 0.12 (0.04 - 0.24) | -8.05 (-10.68 to -5.35) |
| Montenegro | 138 (104 - 181) | 27.58 (20.77 - 36.44) | 0 (0 - 1) | 0.09 (0.03 - 0.19) | -6.59 (-9.32 to -3.78) |
| Poland | 5,734 (5,368 - 6,103) | 20.10 (18.74 - 21.46) | 127 (14 - 440) | 0.35 (0.04 - 1.25) | -2.30 (-4.62 - 0.07) |
| Romania | 31,382 (29,374 - 33,223) | 198.25 (185.39 - 209.97) | 55 (7 - 195) | 0.49 (0.07 - 1.75) | -6.17 (-9.04 to -3.21) |
| Serbia | 2,845 (2,242 - 4,004) | 41.82 (32.85 - 59.18) | 7 (3 - 15) | 0.12 (0.05 - 0.26) | -7.13 (-9.61 to -4.59) |
| Slovakia | 2,760 (2,462 - 3,076) | 67.41 (59.77 - 75.51) | 30 (9 - 72) | 0.89 (0.25 - 2.13) | -3.34 (-5.78 to -0.83) |
| Slovenia | 425 (390 - 460) | 33.48 (30.58 - 36.69) | 4 (1 - 11) | 0.25 (0.05 - 0.77) | -4.44 (-7.01 to -1.79) |
| Belarus | 3,467 (2,947 - 4,063) | 48.12 (40.71 - 56.60) | 258 (65 - 613) | 3.77 (0.95 - 8.81) | -7.13 (-8.17 to -6.08) |
| Estonia | 412 (374 - 453) | 36.39 (32.91 - 40.15) | 3 (1 - 8) | 0.28 (0.06 - 0.78) | -6.29 (-8.86 to -3.64) |
| Latvia | 582 (537 - 629) | 30.15 (27.66 - 32.75) | 2 (0 - 8) | 0.13 (0.01 - 0.66) | -5.96 (-9.24 to -2.56) |
| Lithuania | 641 (580 - 700) | 22.49 (20.30 - 24.66) | 149 (65 - 282) | 7.79 (3.41 - 14.84) | -1.90 (-2.76 to -1.03) |
| Moldova | 5,402 (4,734 - 6,191) | 137.56 (120.49 - 157.71) | 121 (40 - 247) | 6.91 (2.27 - 14.16) | -3.89 (-5.40 to -2.35) |
| Russia | 63,630 (60,891 - 66,541) | 62.05 (59.35 - 64.92) | 2,462 (628 - 5,538) | 2.54 (0.64 - 5.71) | -5.42 (-6.69 to -4.14) |
| Ukraine | 15,543 (13,398 - 17,807) | 45.43 (38.89 - 52.25) | 401 (197 - 677) | 1.65 (0.82 - 2.65) | -3.83 (-5.45 to -2.18) |
| Brunei | 49 (41 - 58) | 17.93 (15.64 - 20.81) | 0 (0 - 0) | 0.02 (0.00 - 0.10) | -3.30 (-7.53 - 1.13) |
| Japan | 14,595 (13,687 - 15,429) | 14.03 (13.34 - 14.65) | 28 (1 - 160) | 0.01 (0.00 - 0.07) | -5.61 (-9.49 to -1.57) |

| location | Num_1990 | DALY_1990 | Num_2021 | DALY_2021 | EAPC_DALY |
|---|---|---|---|---|---|
| South Korea | 6,101 (4,942 - 7,526) | 19.08 (15.38 - 23.49) | 1 (1 - 1) | 0.00 (0.00 - 0.00) | -7.99 (-12.75 to -2.96) |
| Singapore | 864 (803 - 929) | 38.27 (35.62 - 41.17) | 4 (0 - 13) | 0.07 (0.01 - 0.24) | -5.66 (-8.94 to -2.27) |
| Australia | 1,225 (1,141 - 1,312) | 8.99 (8.32 - 9.67) | 9 (2 - 19) | 0.03 (0.01 - 0.08) | -5.10 (-8.54 to -1.52) |
| New Zealand | 617 (569 - 667) | 20.46 (18.84 - 22.22) | 3 (1 - 6) | 0.06 (0.02 - 0.15) | -5.59 (-8.91 to -2.14) |
| Andorra | 3 (2 - 5) | 10.33 (7.30 - 14.79) | 0 (0 - 0) | 0.13 (0.07 - 0.23) | -4.64 (-6.82 to -2.40) |
| Austria | 389 (363 - 413) | 5.86 (5.44 - 6.25) | 15 (8 - 26) | 0.14 (0.07 - 0.23) | -3.97 (-5.85 to -2.07) |
| Belgium | 973 (911 - 1,035) | 11.05 (10.34 - 11.77) | 128 (45 - 267) | 0.88 (0.31 - 1.86) | -2.08 (-3.56 to -0.58) |
| Cyprus | 64 (50 - 84) | 10.44 (8.34 - 13.61) | 2 (1 - 3) | 0.15 (0.07 - 0.24) | -5.23 (-7.14 to -3.29) |
| Denmark | 457 (426 - 488) | 9.87 (9.14 - 10.60) | 10 (2 - 27) | 0.12 (0.02 - 0.35) | -3.91 (-6.35 to -1.40) |
| Finland | 581 (541 - 618) | 11.65 (10.85 - 12.42) | 11 (3 - 27) | 0.21 (0.06 - 0.49) | -6.06 (-7.87 to -4.21) |
| France | 4,224 (3,937 - 4,492) | 7.66 (7.21 - 8.12) | 293 (104 - 597) | 0.35 (0.13 - 0.70) | -3.25 (-4.96 to -1.51) |
| Germany | 5,613 (5,281 - 5,918) | 8.65 (8.17 - 9.13) | 64 (22 - 149) | 0.05 (0.02 - 0.12) | -4.04 (-6.80 to -1.21) |
| Greece | 611 (573 - 647) | 7.70 (7.21 - 8.22) | 4 (1 - 12) | 0.03 (0.00 - 0.08) | -2.37 (-6.10 - 1.52) |
| Iceland | 33 (30 - 36) | 12.91 (11.75 - 14.24) | 19 (10 - 29) | 4.73 (2.36 - 7.23) | -3.09 (-3.81 to -2.36) |
| Ireland | 546 (516 - 579) | 17.13 (16.12 - 18.21) | 27 (6 - 77) | 0.56 (0.12 - 1.59) | -4.17 (-6.01 to -2.30) |
| Israel | 438 (402 - 475) | 8.89 (8.17 - 9.62) | 19 (9 - 31) | 0.16 (0.08 - 0.28) | -3.78 (-5.91 to -1.61) |
| Italy | 3,562 (3,399 - 3,733) | 9.98 (9.49 - 10.44) | 132 (67 - 220) | 0.19 (0.10 - 0.33) | -3.97 (-5.87 to -2.03) |
| Luxembourg | 26 (24 - 28) | 7.78 (7.11 - 8.52) | 1 (1 - 2) | 0.19 (0.09 - 0.32) | -4.04 (-6.03 to -2.01) |
| Malta | 36 (33 - 40) | 11.58 (10.46 - 12.86) | 2 (1 - 4) | 0.41 (0.20 - 0.70) | -2.70 (-4.68 to -0.69) |
| Netherlands | 1,192 (1,107 - 1,275) | 9.42 (8.71 - 10.07) | 64 (10 - 197) | 0.32 (0.05 - 0.97) | -3.32 (-5.36 to -1.24) |
| Norway | 532 (488 - 573) | 10.54 (9.90 - 11.24) | 11 (2 - 33) | 0.13 (0.02 - 0.40) | -5.24 (-7.33 to -3.10) |

| location | Num_1990 | DALY_1990 | Num_2021 | DALY_2021 | EAPC_DALY |
|---|---|---|---|---|---|
| Portugal | 1,421 (1,334 - 1,506) | 20.46 (19.04 - 21.89) | 81 (10 - 270) | 0.53 (0.06 - 1.76) | -3.72 (-5.54 to -1.86) |
| Spain | 2,751 (2,583 - 2,912) | 9.46 (8.92 - 10.04) | 179 (47 - 480) | 0.29 (0.07 - 0.78) | -3.28 (-5.09 to -1.43) |
| Sweden | 912 (846 - 976) | 9.04 (8.52 - 9.60) | 105 (29 - 241) | 0.72 (0.20 - 1.68) | -4.23 (-5.33 to -3.11) |
| Switzerland | 705 (654 - 757) | 11.38 (10.60 - 12.29) | 20 (10 - 33) | 0.18 (0.09 - 0.31) | -5.20 (-7.05 to -3.31) |
| UK | 7,933 (7,510 - 8,362) | 13.79 (13.18 - 14.44) | 360 (184 - 605) | 0.45 (0.23 - 0.76) | -3.86 (-5.92 to -1.76) |
| Argentina | 11,056 (10,327 - 11,861) | 33.32 (31.13 - 35.74) | 823 (334 - 1,677) | 2.20 (0.88 - 4.57) | -2.11 (-4.25 - 0.08) |
| Chile | 7,179 (6,721 - 7,602) | 51.44 (48.33 - 54.32) | 67 (19 - 147) | 0.46 (0.13 - 1.02) | -7.11 (-9.54 to -4.61) |
| Uruguay | 891 (821 - 965) | 32.03 (29.48 - 34.73) | 29 (14 - 51) | 1.11 (0.53 - 1.95) | -4.18 (-6.52 to -1.78) |
| Canada | 2,688 (2,511 - 2,843) | 11.32 (10.63 - 11.99) | 135 (32 - 339) | 0.33 (0.08 - 0.85) | -3.58 (-5.41 to -1.71) |
| USA | 28,472 (27,038 - 29,822) | 11.89 (11.32 - 12.43) | 3,013 (1,349 - 5,468) | 1.02 (0.46 - 1.87) | -2.69 (-4.06 to -1.30) |
| Antigua and Barbuda | 14 (12 - 17) | 23.97 (20.27 - 28.22) | 0 (0 - 0) | 0.17 (0.05 - 0.81) | -1.76 (-5.22 - 1.82) |
| Bahamas | 108 (90 - 132) | 42.84 (35.76 - 51.77) | 0 (0 - 2) | 0.17 (0.05 - 0.80) | -3.04 (-6.47 - 0.52) |
| Barbados | 55 (47 - 64) | 26.30 (22.03 - 30.38) | 0 (0 - 1) | 0.14 (0.04 - 0.72) | -2.50 (-5.95 - 1.07) |
| Belize | 231 (205 - 262) | 79.30 (70.44 - 89.57) | 1 (0 - 4) | 0.21 (0.06 - 0.99) | -4.78 (-8.08 to -1.36) |
| Cuba | 2,729 (2,515 - 2,920) | 30.57 (28.13 - 32.77) | 12 (4 - 58) | 0.14 (0.04 - 0.67) | -2.84 (-6.18 - 0.61) |
| Dominica | 24 (19 - 29) | 28.00 (22.53 - 34.16) | 0 (0 - 0) | 0.28 (0.08 - 1.39) | -0.15 (-3.59 - 3.41) |
| Dominican Republic | 7,692 (6,221 - 9,173) | 74.71 (60.60 - 88.79) | 15 (4 - 70) | 0.14 (0.04 - 0.68) | -5.51 (-8.82 to -2.08) |
| Grenada | 57 (47 - 69) | 51.10 (42.39 - 61.55) | 0 (0 - 1) | 0.18 (0.06 - 0.86) | -3.20 (-6.68 - 0.42) |
| Guyana | 708 (590 - 835) | 60.57 (50.77 - 71.09) | 2 (1 - 11) | 0.31 (0.10 - 1.54) | -2.30 (-5.76 - 1.28) |

| location | Num_1990 | DALY_1990 | Num_2021 | DALY_2021 | EAPC_DALY |
|---|---|---|---|---|---|
| Haiti | 36,103 (28,615 - 45,382) | 315.48 (251.32 - 393.54) | 120 (33 - 601) | 0.75 (0.21 - 3.78) | -5.02 (-8.19 to -1.75) |
| Jamaica | 696 (613 - 795) | 25.76 (22.74 - 29.32) | 2 (1 - 8) | 0.09 (0.03 - 0.43) | -3.81 (-7.11 to -0.40) |
| Saint Lucia | 49 (40 - 58) | 30.17 (25.30 - 35.60) | 0 (0 - 1) | 0.14 (0.04 - 0.69) | -3.04 (-6.41 - 0.45) |
| Saint Vincent and the Grenadines | 43 (36 - 54) | 36.47 (30.10 - 44.57) | 0 (0 - 1) | 0.14 (0.04 - 0.68) | -3.74 (-7.09 to -0.28) |
| Suriname | 272 (214 - 329) | 63.19 (50.02 - 76.29) | 1 (0 - 5) | 0.22 (0.06 - 1.13) | -4.01 (-7.26 to -0.65) |
| Trinidad and Tobago | 398 (339 - 461) | 34.87 (29.85 - 40.23) | 1 (0 - 5) | 0.13 (0.04 - 0.62) | -3.80 (-7.17 to -0.30) |
| Bolivia (Plurinational State of) | 33,780 (24,290 - 43,219) | 324.59 (234.40 - 413.40) | 328 (75 - 798) | 2.82 (0.64 - 6.86) | -5.54 (-8.08 to -2.94) |
| Ecuador | 13,685 (12,251 - 15,199) | 99.53 (89.38 - 110.26) | 1,443 (285 - 3,868) | 9.08 (1.79 - 24.30) | -2.29 (-3.81 to -0.74) |
| Peru | 74,240 (63,584 - 87,237) | 249.44 (214.17 - 292.42) | 15,003 (4,612 - 26,755) | 45.38 (13.94 - 81.05) | -4.21 (-5.50 to -2.90) |
| Colombia | 31,634 (27,375 - 36,151) | 74.01 (64.21 - 84.41) | 1,194 (266 - 3,026) | 3.36 (0.74 - 8.48) | -3.30 (-5.05 to -1.51) |
| Costa Rica | 1,407 (1,289 - 1,542) | 36.82 (33.87 - 40.23) | 15 (5 - 31) | 0.48 (0.15 - 1.01) | -3.80 (-6.36 to -1.18) |
| El Salvador | 6,997 (5,779 - 8,252) | 88.01 (72.91 - 103.62) | 47 (9 - 124) | 0.81 (0.16 - 2.12) | -5.20 (-8.06 to -2.25) |
| Guatemala | 26,663 (23,994 - 29,766) | 177.83 (160.75 - 197.87) | 4,474 (1,691 - 9,065) | 30.89 (11.67 - 62.58) | -0.24 (-1.64 - 1.18) |
| Honduras | 9,333 (7,712 - 11,117) | 112.03 (92.97 - 132.93) | 1,621 (464 - 3,765) | 15.55 (4.45 - 36.22) | -3.09 (-4.09 to -2.08) |
| Mexico | 141,400 (128,181 - 158,640) | 119.40 (108.40 - 133.86) | 5,936 (3,035 - 10,170) | 6.14 (3.15 - 10.49) | -3.95 (-5.23 to -2.64) |
| Nicaragua | 8,809 (7,489 - 10,313) | 132.68 (113.10 - 155.34) | 431 (148 - 887) | 6.94 (2.38 - 14.26) | -3.71 (-6.02 to -1.33) |
| Panama | 1,284 (1,090 - | 46.24 (39.39 - | 664 (149 - | 18.83 (4.22 - | 0.12 (-1.28 - |

| location | Num_1990 | DALY_1990 | Num_2021 | DALY_2021 | EAPC_DALY |
|---|---|---|---|---|---|
| | 1,488) | 53.46) | 1,576) | 44.63) | 1.54) |
| Venezuela | 13,429 (12,492 - 14,407) | 53.18 (49.58 - 56.98) | 1,182 (618 - 2,005) | 5.27 (2.76 - 8.96) | -0.76 (-2.51 - 1.03) |
| Brazil | 171,673 (152,464 - 191,924) | 111.58 (99.24 - 124.57) | 12,160 (3,403 - 27,554) | 6.69 (1.88 - 15.25) | -4.45 (-5.83 to -3.06) |
| Paraguay | 3,925 (3,080 - 4,936) | 64.79 (51.06 - 81.14) | 211 (51 - 489) | 3.34 (0.81 - 7.74) | -3.41 (-5.39 to -1.39) |
| Algeria | 30,774 (22,429 - 42,452) | 85.46 (62.90 - 117.25) | 3,091 (1,405 - 5,748) | 7.10 (3.23 - 13.23) | -3.56 (-4.59 to -2.51) |
| Bahrain | 239 (202 - 295) | 39.93 (34.15 - 48.92) | 34 (15 - 68) | 3.88 (1.64 - 7.60) | -1.85 (-3.24 to -0.45) |
| Egypt | 323,648 (267,038 - 399,910) | 367.28 (303.41 - 453.39) | 24,907 (8,185 - 53,369) | 20.56 (6.75 - 43.80) | -5.34 (-6.17 to -4.50) |
| Iran | 73,868 (60,793 - 97,872) | 97.09 (80.14 - 127.97) | 3,935 (1,889 - 6,038) | 6.30 (2.96 - 9.94) | -6.02 (-6.85 to -5.19) |
| Iraq | 33,386 (26,289 - 42,467) | 103.80 (81.91 - 131.58) | 3,935 (1,710 - 7,407) | 9.73 (4.22 - 18.29) | -3.99 (-4.90 to -3.06) |
| Jordan | 4,351 (3,589 - 5,227) | 71.26 (59.18 - 85.29) | 812 (222 - 1,913) | 7.83 (2.13 - 18.41) | -2.62 (-3.71 to -1.53) |
| Kuwait | 745 (654 - 846) | 43.21 (38.10 - 49.01) | 330 (152 - 625) | 12.89 (5.92 - 24.32) | 1.53 (0.21 - 2.87) |
| Lebanon | 3,611 (2,537 - 4,899) | 90.18 (63.90 - 121.95) | 438 (202 - 829) | 10.27 (4.70 - 19.64) | -3.33 (-4.27 to -2.39) |
| Libya | 3,058 (2,283 - 4,127) | 50.11 (37.59 - 67.15) | 363 (150 - 721) | 8.44 (3.47 - 16.99) | -0.40 (-1.71 - 0.92) |
| Morocco | 72,441 (55,276 - 92,458) | 195.69 (149.59 - 249.57) | 3,412 (1,363 - 6,712) | 10.89 (4.34 - 21.47) | -5.07 (-6.08 to -4.05) |
| Palestine | 3,070 (2,329 - 3,936) | 77.38 (59.01 - 98.31) | 446 (203 - 880) | 7.97 (3.63 - 15.49) | -2.86 (-3.90 to -1.80) |
| Oman | 2,418 (1,770 - 3,705) | 74.28 (54.51 - 111.65) | 306 (119 - 620) | 8.18 (3.18 - 16.46) | -2.13 (-3.74 to -0.49) |
| Qatar | 138 (102 - 177) | 27.73 (20.90 - 35.39) | 10 (4 - 23) | 0.59 (0.20 - 1.28) | -3.42 (-5.69 to -1.09) |
| Saudi Arabia | 16,297 (12,431 - 22,055) | 70.47 (54.04 - 94.22) | 1,028 (444 - 1,974) | 3.81 (1.67 - 7.23) | -4.60 (-5.72 to -3.46) |
| Syria | 16,062 (12,115 - 22,428) | 75.32 (56.93 - 104.59) | 669 (264 - 1,375) | 6.38 (2.51 - 13.26) | -1.13 (-2.84 - 0.61) |
| Tunisia | 13,214 (9,015 - 21,173) | 126.76 (86.72 - 202.44) | 557 (232 - 1,084) | 6.34 (2.62 - 12.41) | -5.51 (-6.45 to -4.57) |
| Turkey | 120,682 (91,267 - | 172.97 (131.07 - | 3,353 (1,551 | 5.93 (2.71 - | -6.77 (-7.70 |

| location | Num_1990 | DALY_1990 | Num_2021 | DALY_2021 | EAPC_DALY |
|---|---|---|---|---|---|
| | 162,782) | 232.82) | - 6,476) | 11.46) | to -5.84) |
| United Arab Emirates | 832 (611 - 1,130) | 39.28 (29.42 - 52.18) | 135 (62 - 252) | 3.68 (1.68 - 6.82) | -1.94 (-3.31 to -0.55) |
| Yemen | 64,157 (48,488 - 90,594) | 217.72 (165.59 - 306.43) | 8,358 (3,737 - 15,544) | 18.46 (8.24 - 34.36) | -3.91 (-4.86 to -2.94) |
| Afghanistan | 69,646 (53,087 - 92,776) | 357.58 (274.53 - 476.40) | 14,229 (6,214 - 28,315) | 25.71 (11.31 - 50.82) | -4.25 (-5.28 to -3.22) |
| Bangladesh | 586,056 (491,258 - 692,060) | 300.00 (252.04 - 353.42) | 62,391 (26,978 - 116,637) | 45.62 (19.70 - 85.35) | -4.17 (-5.01 to -3.32) |
| Bhutan | 2,270 (1,525 - 3,059) | 223.36 (148.56 - 301.12) | 223 (94 - 423) | 36.99 (15.66 - 70.13) | -4.29 (-4.94 to -3.63) |
| India | 2,487,129 (1,976,069 - 2,987,872) | 212.61 (169.35 - 255.09) | 674,441 (251,584 - 1,245,583) | 63.46 (23.68 - 117.18) | -2.39 (-3.07 to -1.70) |
| Nepal | 148,205 (120,484 - 179,046) | 415.81 (337.40 - 503.34) | 13,053 (4,762 - 26,676) | 42.60 (15.57 - 86.92) | -4.86 (-5.54 to -4.16) |
| Pakistan | 422,213 (333,746 - 522,095) | 216.83 (171.94 - 267.12) | 209,316 (115,779 - 336,797) | 71.24 (39.38 - 114.63) | -0.67 (-1.64 - 0.32) |
| Angola | 65,088 (49,102 - 84,323) | 307.29 (233.67 - 394.87) | 31,339 (13,972 - 52,095) | 59.66 (27.06 - 97.91) | -4.47 (-4.81 to -4.14) |
| Central African Republic | 21,314 (14,363 - 28,775) | 385.12 (263.89 - 516.44) | 11,802 (5,403 - 19,793) | 139.65 (64.57 - 234.29) | -2.32 (-2.59 to -2.06) |
| Congo (Brazzaville) | 5,657 (3,850 - 7,863) | 141.13 (98.12 - 193.16) | 2,278 (1,053 - 3,701) | 40.17 (18.74 - 64.61) | -3.31 (-3.64 to -2.98) |
| DR Congo | 208,430 (140,829 - 279,452) | 264.73 (181.59 - 351.89) | 81,648 (38,060 - 133,910) | 65.32 (31.07 - 106.45) | -3.47 (-3.84 to -3.10) |
| Equatorial Guinea | 2,275 (1,593 - 3,036) | 254.41 (178.82 - 338.12) | 629 (263 - 1,142) | 38.98 (16.62 - 69.40) | -6.11 (-6.42 to -5.80) |
| Gabon | 2,308 (1,614 - 3,144) | 144.36 (101.92 - 194.85) | 784 (348 - 1,372) | 40.90 (18.30 - 71.07) | -2.86 (-3.19 to -2.52) |

| location | Num_1990 | DALY_1990 | Num_2021 | DALY_2021 | EAPC_DALY |
|---|---|---|---|---|---|
| Burundi | 26,858 (19,324 - 35,220) | 238.09 (172.30 - 307.98) | 11,659 (6,455 - 19,738) | 56.53 (31.84 - 94.08) | -2.57 (-3.46 to -1.67) |
| Comoros | 2,610 (1,994 - 3,410) | 293.27 (222.85 - 381.40) | 538 (336 - 802) | 67.68 (42.32 - 100.67) | -3.37 (-3.96 to -2.77) |
| Djibouti | 1,123 (821 - 1,471) | 164.12 (120.88 - 213.46) | 594 (352 - 924) | 43.20 (25.75 - 66.76) | -2.64 (-3.45 to -1.82) |
| Eritrea | 14,220 (10,799 - 18,250) | 222.49 (169.08 - 282.12) | 6,897 (4,256 - 10,717) | 79.39 (49.28 - 122.09) | -1.59 (-2.28 to -0.89) |
| Ethiopia | 309,971 (232,530 - 391,464) | 302.45 (227.86 - 381.09) | 74,705 (48,180 - 113,221) | 48.33 (31.33 - 72.63) | -4.74 (-5.31 to -4.16) |
| Kenya | 86,975 (71,633 - 111,968) | 195.22 (161.54 - 250.59) | 29,027 (17,745 - 43,881) | 53.35 (32.74 - 79.98) | -1.79 (-2.75 to -0.82) |
| Madagascar | 71,592 (61,451 - 83,189) | 306.15 (263.77 - 354.61) | 25,398 (8,842 - 50,138) | 64.90 (22.57 - 128.07) | -2.49 (-3.37 to -1.61) |
| Malawi | 63,763 (46,286 - 84,955) | 306.01 (224.25 - 403.11) | 20,323 (11,588 - 31,155) | 78.18 (45.18 - 119.60) | -2.63 (-3.42 to -1.84) |
| Mauritius | 478 (438 - 520) | 45.36 (41.71 - 49.33) | 15 (8 - 27) | 2.11 (1.05 - 3.63) | -1.08 (-3.10 - 0.98) |
| Mozambique | 57,385 (42,246 - 84,514) | 220.03 (162.63 - 322.45) | 26,516 (16,345 - 41,267) | 54.39 (33.54 - 83.92) | -2.57 (-3.29 to -1.85) |
| Rwanda | 43,359 (31,976 - 56,058) | 312.96 (232.28 - 405.20) | 10,238 (6,053 - 15,727) | 60.72 (36.34 - 92.94) | -4.02 (-4.80 to -3.24) |
| Seychelles | 46 (40 - 54) | 60.81 (52.30 - 69.95) | 3 (1 - 5) | 3.25 (1.65 - 5.61) | -0.61 (-2.68 - 1.50) |
| Somalia | 49,275 (34,112 - 66,728) | 295.25 (206.45 - 396.05) | 29,483 (17,869 - 47,629) | 70.39 (43.44 - 113.50) | -2.96 (-3.67 to -2.24) |
| Tanzania | 187,324 (147,129 - 230,039) | 357.25 (280.86 - 436.53) | 78,032 (43,713 - 127,517) | 90.30 (50.77 - 147.31) | -3.13 (-3.82 to -2.45) |
| Uganda | 55,522 (38,575 - 79,641) | 144.36 (101.64 - 209.20) | 29,486 (10,685 - 63,387) | 42.85 (15.73 - 91.02) | -1.92 (-2.78 to -1.06) |
| Zambia | 47,959 (37,730 - 61,528) | 292.56 (229.17 - 374.51) | 23,302 (12,903 - | 84.95 (47.44 - 129.55) | -2.55 (-3.08 to -2.02) |

| location | Num_1990 | DALY_1990 | Num_2021 | DALY_2021 | EAPC_DALY |
|---|---|---|---|---|---|
| | | | 35,654) | | |
| Botswana | 2,227 (1,713 - 2,790) | 111.27 (87.55 - 139.63) | 504 (192 - 996) | 21.93 (8.35 - 43.32) | -1.00 (-2.73 - 0.76) |
| Lesotho | 3,651 (2,993 - 4,493) | 149.92 (123.88 - 183.73) | 871 (326 - 1,640) | 44.23 (16.52 - 83.31) | 0.13 (-1.47 - 1.75) |
| Namibia | 2,327 (1,797 - 2,923) | 104.98 (82.19 - 131.81) | 453 (170 - 921) | 17.28 (6.46 - 35.22) | -1.30 (-2.99 - 0.42) |
| South Africa | 87,963 (74,911 - 105,683) | 182.34 (156.03 - 218.28) | 9,425 (3,764 - 19,129) | 19.40 (7.74 - 39.35) | -2.46 (-4.44 to -0.44) |
| Eswatini | 2,030 (1,631 - 2,530) | 142.62 (115.13 - 177.08) | 351 (142 - 691) | 26.20 (10.73 - 51.64) | -1.01 (-2.80 - 0.81) |
| Zimbabwe | 28,869 (24,560 - 33,889) | 165.89 (141.74 - 194.00) | 7,839 (3,075 - 15,448) | 36.94 (14.59 - 72.64) | -0.44 (-2.14 - 1.30) |
| Benin | 31,337 (24,624 - 38,758) | 303.91 (240.43 - 376.72) | 20,604 (11,817 - 31,720) | 88.24 (50.38 - 135.69) | -1.60 (-2.28 to -0.92) |
| Burkina Faso | 85,599 (64,582 - 107,003) | 421.97 (317.48 - 527.51) | 61,302 (35,533 - 93,115) | 145.03 (83.87 - 219.76) | -1.09 (-1.75 to -0.42) |
| Cameroon | 49,930 (38,297 - 65,241) | 238.17 (183.45 - 311.05) | 38,721 (17,661 - 67,201) | 82.03 (37.48 - 141.90) | -1.17 (-1.92 to -0.41) |
| Cape Verde | 485 (356 - 645) | 84.47 (63.49 - 112.13) | 85 (51 - 144) | 19.54 (11.51 - 33.02) | -3.52 (-4.16 to -2.88) |
| Chad | 45,330 (35,822 - 56,017) | 335.14 (264.03 - 411.69) | 46,455 (29,176 - 69,975) | 123.85 (77.87 - 185.79) | -1.41 (-1.96 to -0.85) |
| Côte d'Ivoire | 71,504 (54,851 - 94,995) | 290.76 (224.68 - 386.47) | 24,357 (12,461 - 40,327) | 56.47 (28.85 - 93.48) | -1.70 (-2.60 to -0.79) |
| The Gambia | 4,657 (3,702 - 5,857) | 239.50 (191.09 - 300.18) | 1,755 (1,116 - 2,595) | 52.81 (33.91 - 78.01) | -3.20 (-3.81 to -2.59) |
| Ghana | 42,601 (32,848 - 54,702) | 160.61 (124.97 - 204.40) | 20,663 (11,086 - 34,485) | 47.82 (25.76 - 78.33) | -1.31 (-2.06 to -0.56) |
| Guinea | 61,822 (47,081 - 78,635) | 494.59 (376.08 - 631.36) | 20,812 (11,754 - 31,806) | 93.23 (53.17 - 141.74) | -3.13 (-3.77 to -2.50) |
| Guinea-Bissau | 5,416 (4,111 - 6,911) | 280.02 (213.82 - 355.55) | 1,444 (880 - 2,195) | 47.46 (29.48 - 71.57) | -3.65 (-4.28 to -3.01) |
| Liberia | 19,245 (14,459 - 25,358) | 392.95 (292.60 - 518.71) | 3,496 (2,039 - 5,558) | 47.81 (27.97 - 75.27) | -4.97 (-5.80 to -4.14) |

| location | Num_1990 | DALY_1990 | Num_2021 | DALY_2021 | EAPC_DALY |
|---|---|---|---|---|---|
| Mali | 36,781 (28,890 - 46,838) | 196.56 (152.92 - 251.43) | 33,346 (19,853 - 49,754) | 71.38 (42.92 - 106.39) | -1.23 (-2.06 to -0.40) |
| Mauritania | 7,293 (5,815 - 8,848) | 189.39 (152.03 - 228.74) | 2,966 (1,857 - 4,639) | 48.25 (30.49 - 74.64) | -2.48 (-3.11 to -1.85) |
| Niger | 101,553 (76,222 - 132,982) | 565.79 (419.80 - 742.20) | 54,213 (31,536 - 82,902) | 104.70 (60.72 - 160.64) | -3.80 (-4.41 to -3.18) |
| Nigeria | 805,605 (657,267 - 956,300) | 464.90 (376.92 - 554.40) | 507,757 (290,920 - 780,237) | 134.80 (76.83 - 206.92) | -1.94 (-2.54 to -1.34) |
| São Tomé and Príncipe | 484 (384 - 583) | 234.32 (186.53 - 281.45) | 69 (39 - 121) | 31.60 (18.11 - 54.36) | -4.61 (-5.24 to -3.98) |
| Senegal | 34,928 (28,134 - 41,987) | 229.43 (185.34 - 276.49) | 13,363 (6,666 - 24,067) | 60.48 (30.45 - 107.81) | -2.35 (-3.07 to -1.62) |
| Sierra Leone | 35,893 (26,447 - 47,678) | 420.50 (307.97 - 557.16) | 10,022 (5,684 - 16,724) | 75.41 (42.99 - 125.39) | -3.81 (-4.38 to -3.23) |
| Togo | 17,067 (13,376 - 21,439) | 242.26 (190.74 - 303.74) | 11,377 (6,149 - 18,054) | 102.87 (56.07 - 161.85) | -1.54 (-1.99 to -1.08) |
| American Samoa | 41 (34 - 48) | 52.69 (44.50 - 62.26) | 1 (0 - 1) | 1.68 (0.83 - 2.86) | -3.31 (-5.17 to -1.41) |
| Bermuda | 6 (6 - 7) | 13.59 (11.76 - 15.74) | 0 (0 - 0) | 0.03 (0.01 - 0.17) | -5.23 (-8.44 to -1.91) |
| Cook Islands | 26 (21 - 33) | 126.27 (100.73 - 155.42) | 0 (0 - 1) | 2.49 (1.24 - 4.24) | -7.07 (-8.42 to -5.70) |
| Greenland | 20 (16 - 25) | 36.56 (29.38 - 45.81) | 1 (0 - 1) | 1.33 (0.63 - 2.35) | -4.20 (-5.71 to -2.67) |
| Guam | 61 (52 - 72) | 36.73 (31.63 - 42.46) | 2 (1 - 3) | 1.35 (0.63 - 2.31) | -1.93 (-4.11 - 0.29) |
| Monaco | 3 (3 - 4) | 10.50 (7.73 - 14.06) | 0 (0 - 0) | 0.58 (0.28 - 0.98) | -2.70 (-4.39 to -0.99) |
| Nauru | 22 (17 - 27) | 136.46 (108.37 - 168.32) | 1 (0 - 2) | 8.02 (3.66 - 14.12) | -1.46 (-3.59 - 0.73) |
| Niue | 2 (2 - 3) | 93.80 (74.61 - 116.25) | 0 (0 - 0) | 21.51 (11.03 - 35.65) | 0.62 (-0.89 - 2.15) |

| location | Num_1990 | DALY_1990 | Num_2021 | DALY_2021 | EAPC_DALY |
|---|---|---|---|---|---|
| Northern Mariana Islands | 17 (13 - 22) | 32.80 (25.38 - 42.03) | 0 (0 - 1) | 0.87 (0.42 - 1.53) | -3.14 (-5.22 to -1.03) |
| Palau | 17 (13 - 22) | 116.72 (86.76 - 150.83) | 0 (0 - 1) | 5.05 (2.36 - 8.64) | -1.85 (-3.84 - 0.17) |
| Puerto Rico | 773 (722 - 826) | 23.91 (22.30 - 25.55) | 1 (0 - 7) | 0.08 (0.02 - 0.36) | -4.66 (-8.09 to -1.10) |
| Saint Kitts and Nevis | 22 (20 - 25) | 50.26 (45.06 - 56.31) | 0 (0 - 0) | 0.25 (0.08 - 1.20) | -2.80 (-6.15 - 0.68) |
| San Marino | 1 (1 - 2) | 8.37 (6.53 - 11.04) | 0 (0 - 0) | 0.12 (0.06 - 0.22) | -4.89 (-6.96 to -2.79) |
| Tokelau | 2 (1 - 2) | 89.46 (67.02 - 113.91) | 0 (0 - 0) | 10.78 (5.10 - 18.92) | -3.45 (-4.51 to -2.37) |
| Tuvalu | 44 (34 - 57) | 278.60 (215.08 - 358.35) | 0 (0 - 1) | 3.62 (1.77 - 6.25) | -5.90 (-7.68 to -4.08) |
| Virgin Islands | 20 (15 - 25) | 18.50 (14.12 - 23.17) | 0 (0 - 0) | 0.04 (0.01 - 0.20) | -5.10 (-8.46 to -1.61) |
| South Sudan | 34,748 (26,646 - 45,975) | 314.85 (241.75 - 414.94) | 17,570 (10,189 - 31,389) | 106.49 (62.09 - 187.90) | -1.92 (-2.64 to -1.19) |
| Sudan | 99,994 (73,431 - 136,865) | 265.64 (194.26 - 365.76) | 8,607 (3,780 - 16,177) | 15.88 (6.97 - 29.79) | -4.96 (-5.96 to -3.95) |
